# Supplementary material for: Chromosome-scale assembly and analysis of biomass crop Miscanthus lutarioriparius genome
Source: Nat Commun. 2021 Apr 28;12:2458. doi: 10.1038/s41467-021-22738-4 (PMC8080599; doi:10.1038/s41467-021-22738-4)
Supplement: Supplementary file 1 — Supplementary Information [file 41467_2021_22738_MOESM1_ESM.pdf]

Chromosome-scale assembly and analysis of biomass crop *Miscanthus lutarioriparius* genome

Miao and Feng *et al.*

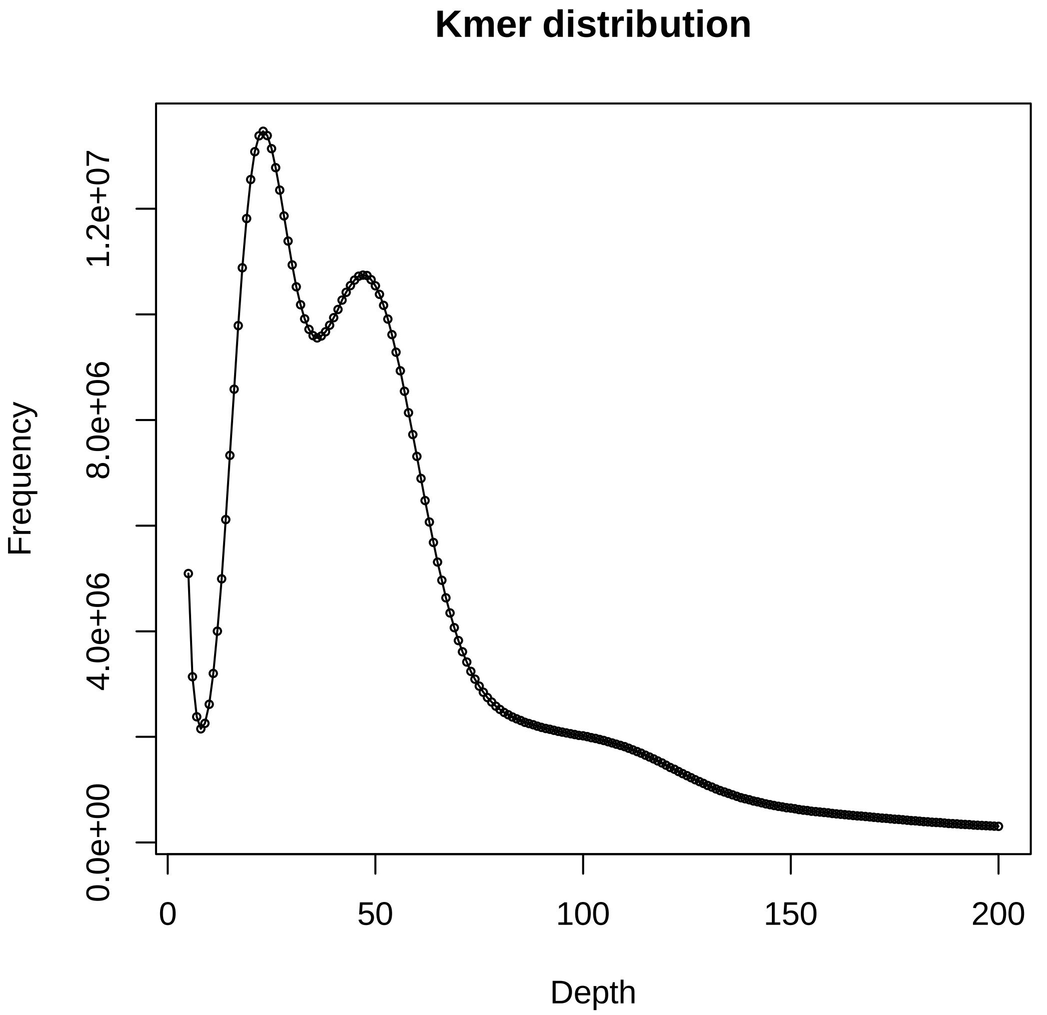

Supplementary Fig. 1. The 17-mer frequency distribution using NGS short reads.

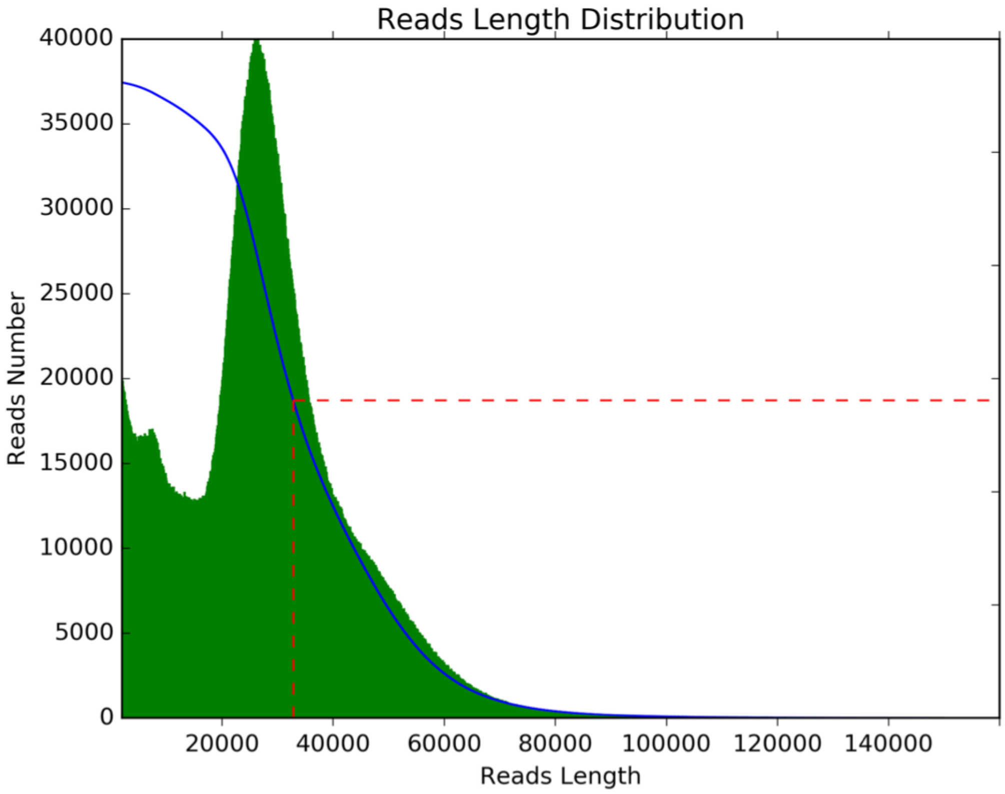

Supplementary Fig. 2. The length distribution of filtered Oxford Nanopore reads.

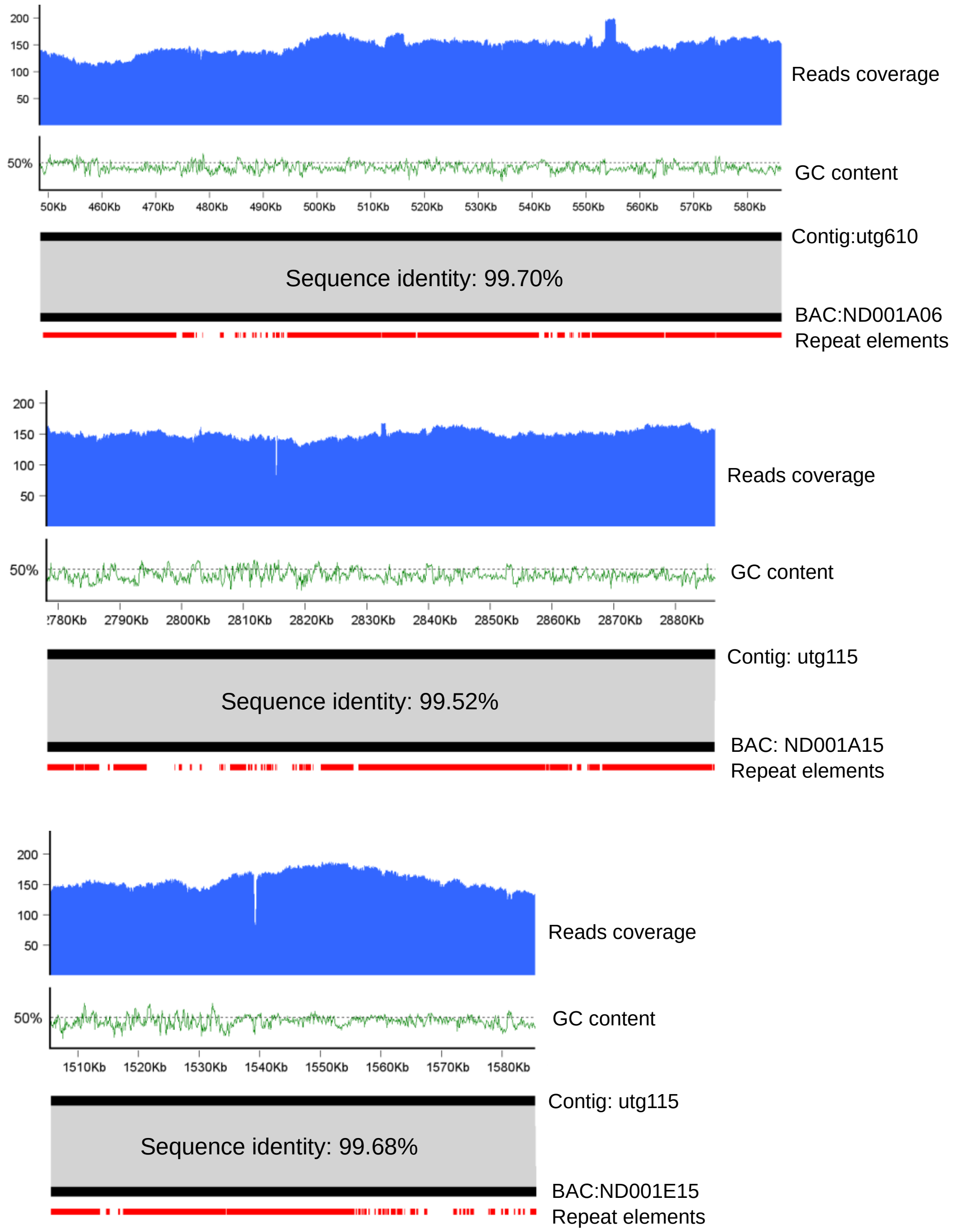

Supplementary Fig. 3. Sequence comparison between Nanopore contigs and BAC sequences of *M. lutarioriparius.* Reads coverage was calculated by mapping the filtered Oxford Nanopore reads against the polished contigs of *M. lutarioriparius* and using a 100 bp window size. GC content was calculated using a 100 bp window size. The repeat elements of BAC sequences were identified using RepeatMasker with *Miscanthus* specific repeats database generated in present study. The sequence comparisons were performed by MUMer 3.2 software. The sequence identity was calculated using the results generated by MUMer 3.2 software.

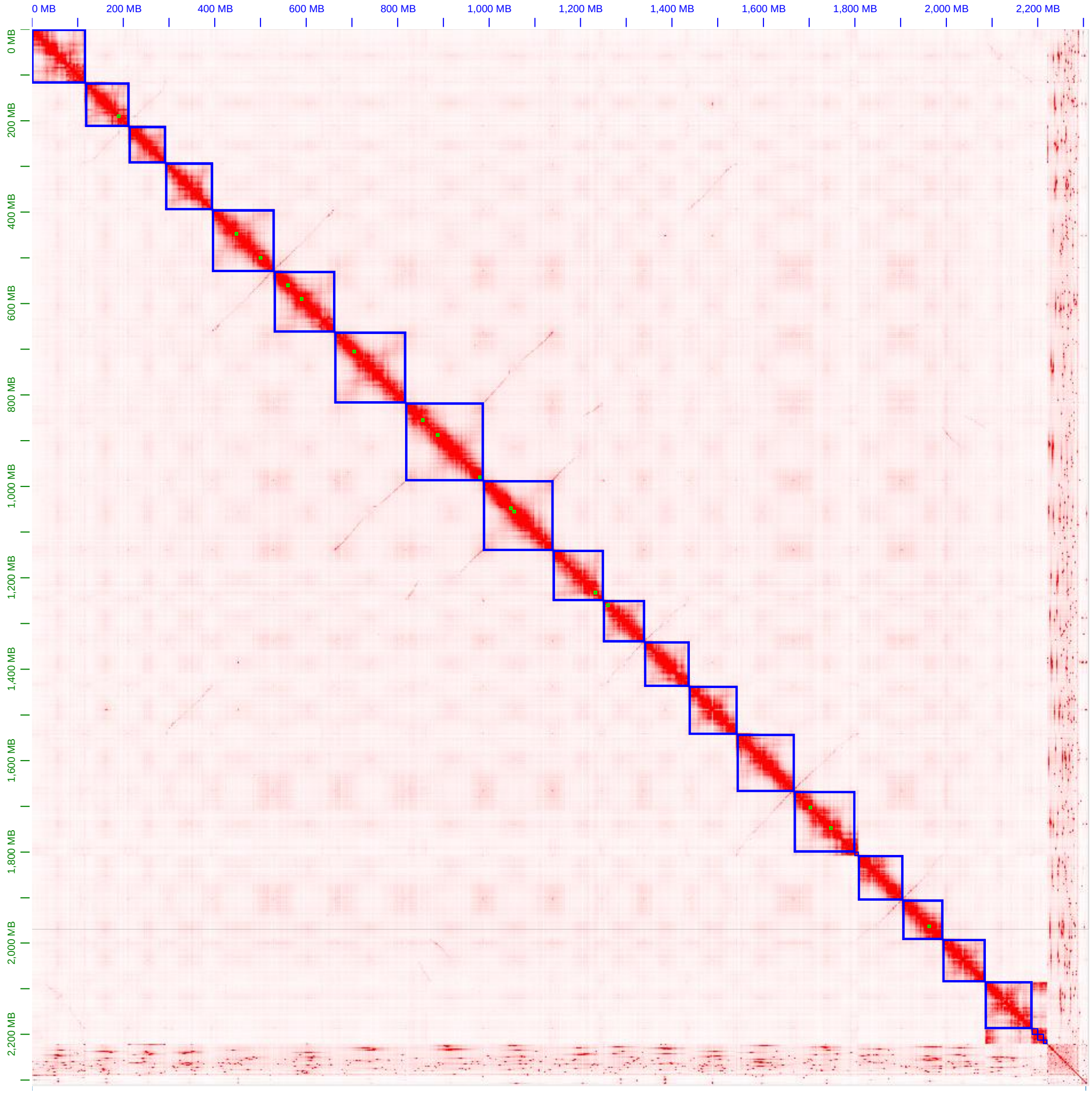

Supplementary Fig. 4. Interaction frequency distribution of Hi-C links among chromosomes. The Hi-C version of *M. lutarioriparius* genome assembly generated by 3d-dna pipeline.

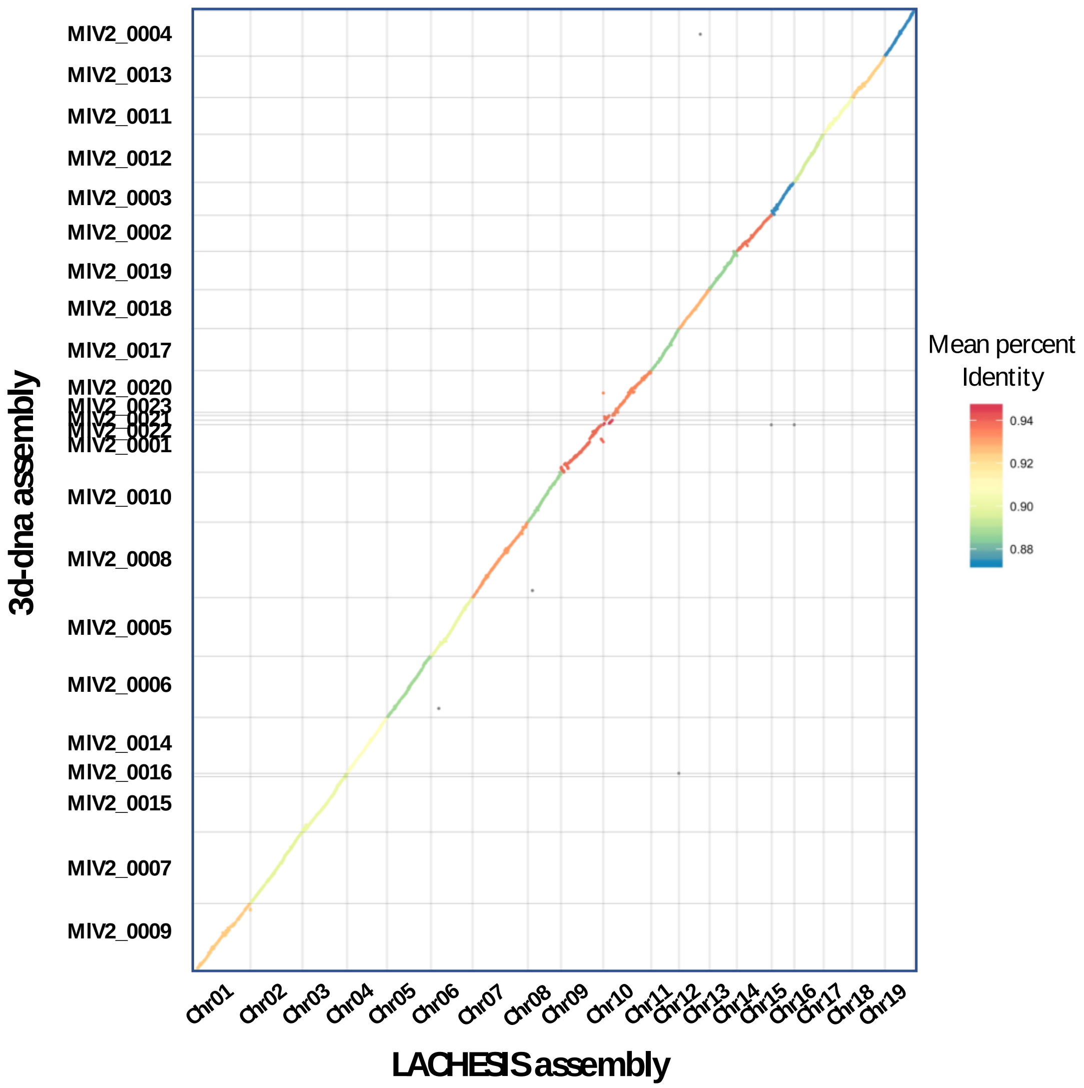

Supplementary Fig. 5. Comparison of sequences identity of chromosome anchoring results generated by LACHESIS and 3d-dna software. The whole genome sequence comparison was performed using minmap2 (version 2.16) software.

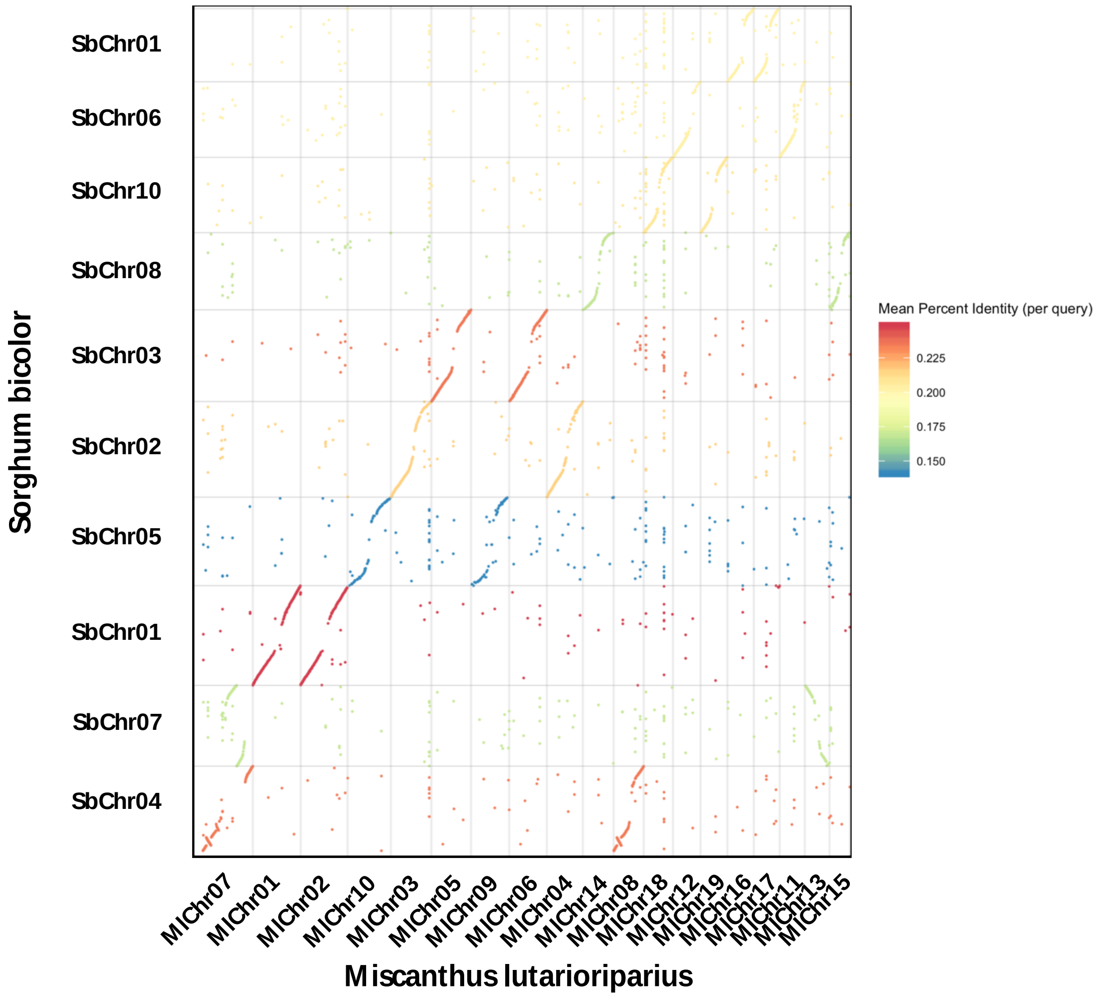

Supplementary Fig. 6. Comparison of whole genome sequence similarity between *M. lutarioriparius* LACHESIS genome assembly (final genome assembly) and sorghum genome. The sequence comparison was performed using minmap2 (version 2.16) software.

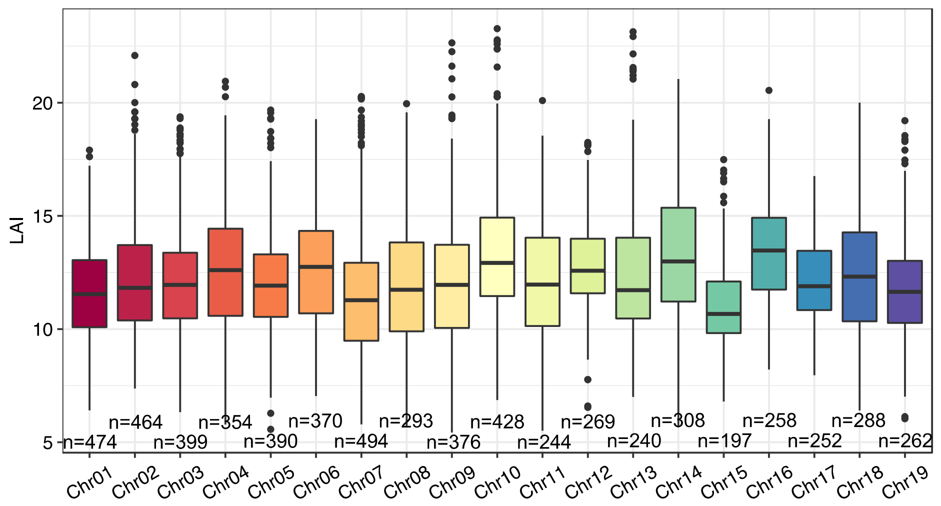

Supplementary Fig. 7. Boxplot of LTR Assembly Index (LAI) for 19 chromosomes. In each boxplot, the center line indicates the median, the lower and upper hinges represent the first and third quartiles, the upper whisker extends to the largest value less than 1.5× the interquartile range (IQR), the lower whisker extends to the smallest value at most 1.5× the IQR, the black point represent outlier. The number of data points used for plotting is shown at the bottom.

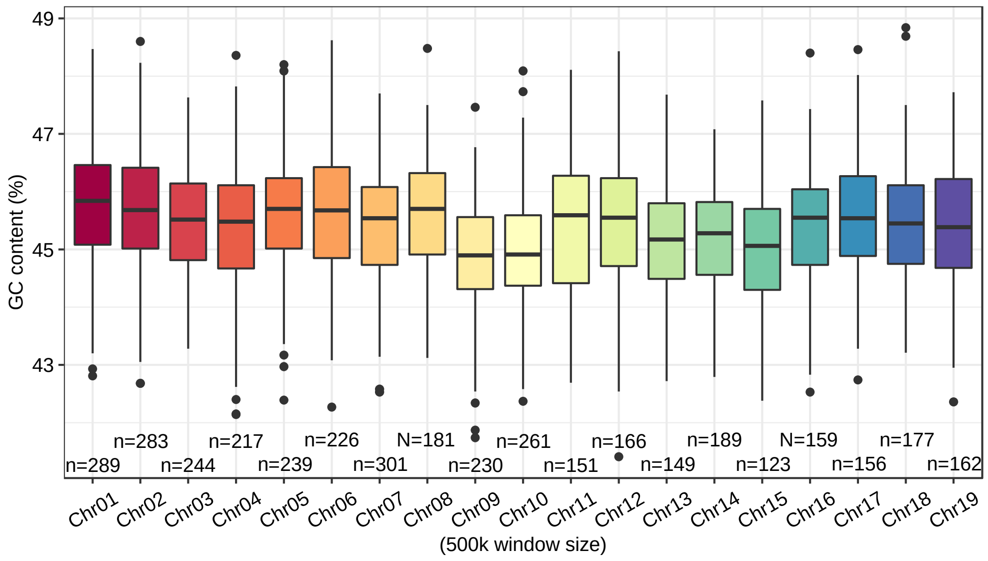

Supplementary Fig. 8. GC content of each chromosome of *M. lutarioriparius*. In each boxplot, the center line indicates the median, the lower and upper hinges represent the first and third quartiles, the upper whisker extends to the largest value less than 1.5× the interquartile range (IQR), the lower whisker extends to the smallest value at most 1.5× the IQR, the larger black points represent outliers. The data points are indicated with smaller black points. The number of data points used for plotting is shown at the bottom.

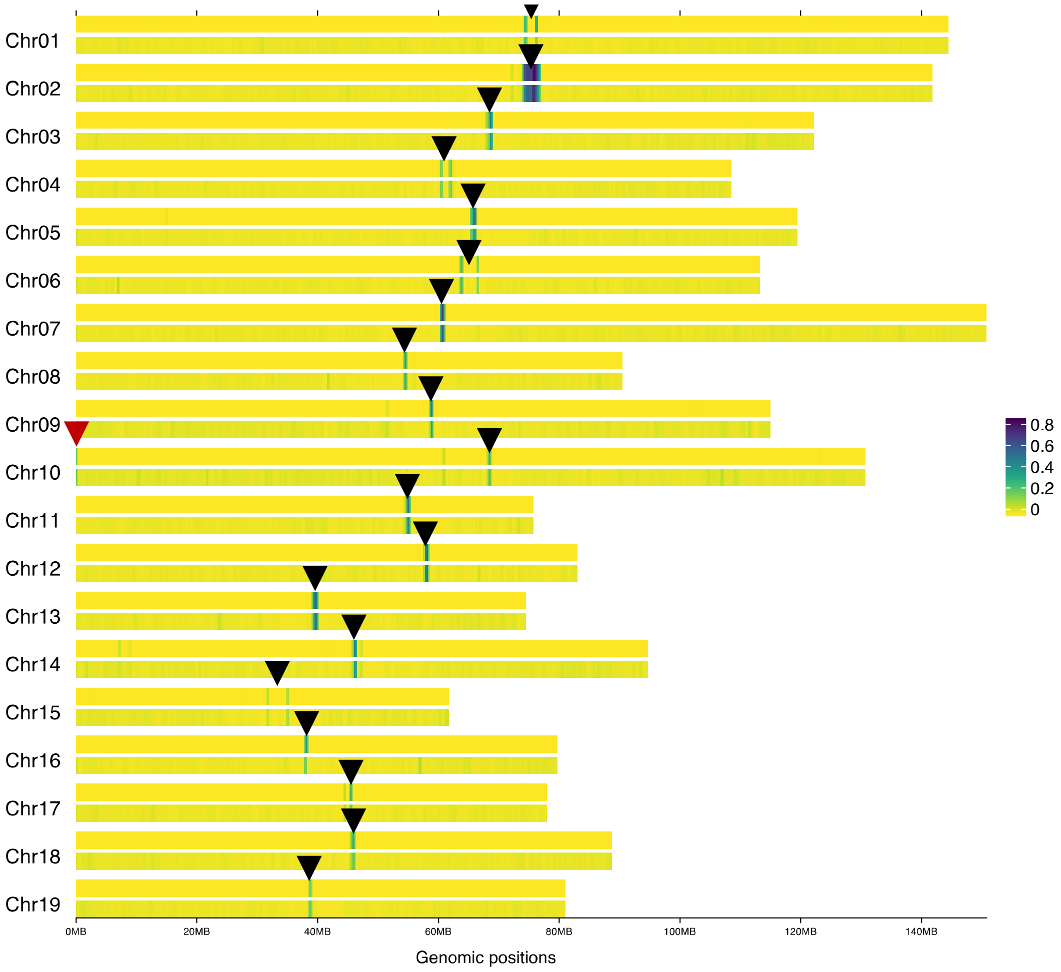

Supplementary Fig. 9. Potential centromere and telomere position of *M. lutarioriparius* genome. The heatmap tracks for each chromosome represent the density of tandem repeats (non-overlap window size = 500 kb) identified by Tandem Repeat Finder (upper track) and the simple sequence repeats identified by RepeatMasker (lower track). Black and red inverted triangles were used to indicate the potential locations of centromere and telomere, respectively.

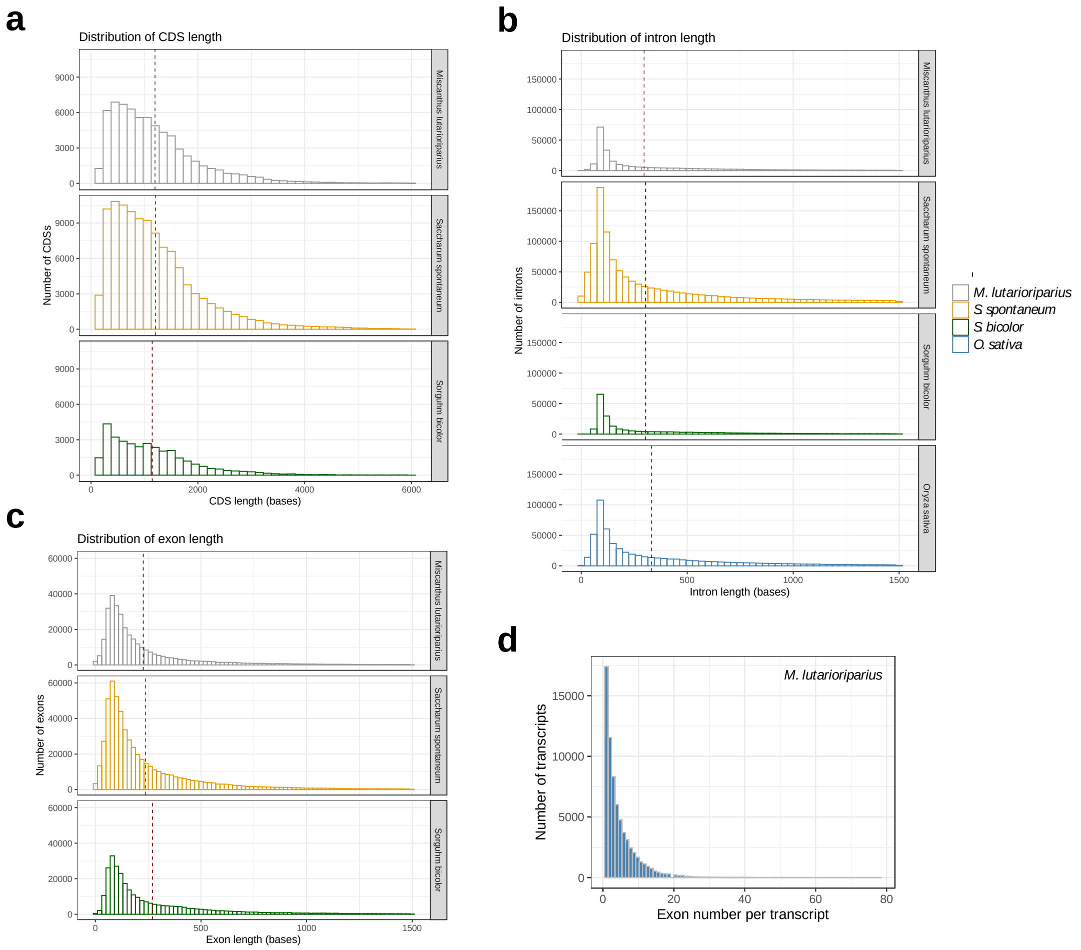

Supplementary Fig. 10. Statistic of gene prediction. The length distribution of protein-coding sequence (CDS) (**a**), intron (**b**) and exon (**c**) of *M. lutarioriparius, S. spontaneum* and *S. bicolor*. **d** the exon number distribution of *M. lutarioriparius.*

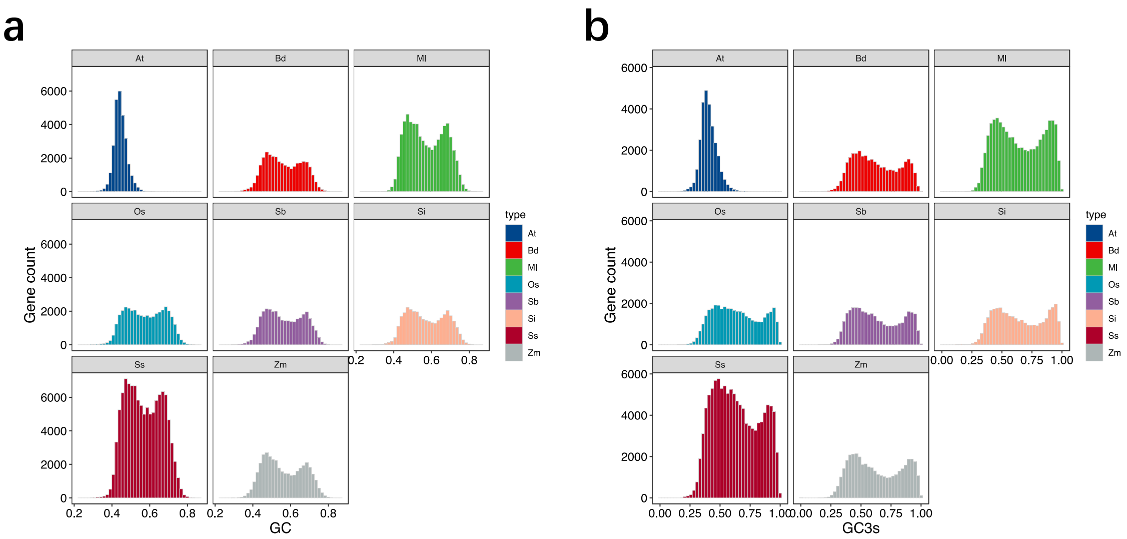

Supplementary Fig. 11. Comparison of GC content distribution (a) and GC3s content distribution (b) of whole genome coding sequences (CDS) for 8 species. At: *Arabidopsis thaliana*. Bd: *Brachypodium distachyon*. Ml: *Miscanthus lutarioriparius*. Os: *Oryza sativa*. Sb: *Sorghum bicolor*. Si: *Seteria italica*. Ss: *Saccharum spontaneum*. Zm: *Zea mays*

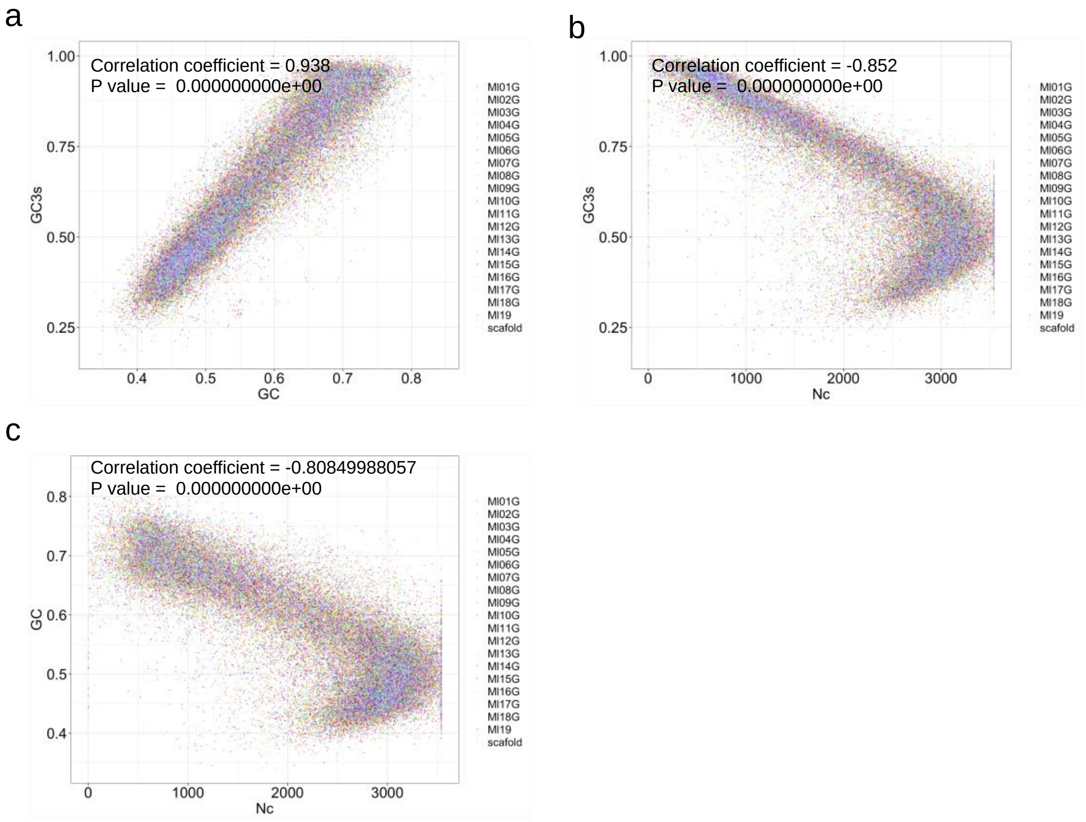

Supplementary Fig. 12. The correlation analysis of GC3s, GC content and effective number of codons (Nc) for *M. lutarioriparius* protein-coding genes. Pearson's rho rank correlation coefficient was computed using the R function rcorr within Hmisc package. The GC3s of *M. lutarioriparius* CDS has a strong positive correlation with GC content (**a**). The effective number of codons (Nc) of *M. lutarioriparius* CDS has negative correlation with GC and GC3s, respectively.

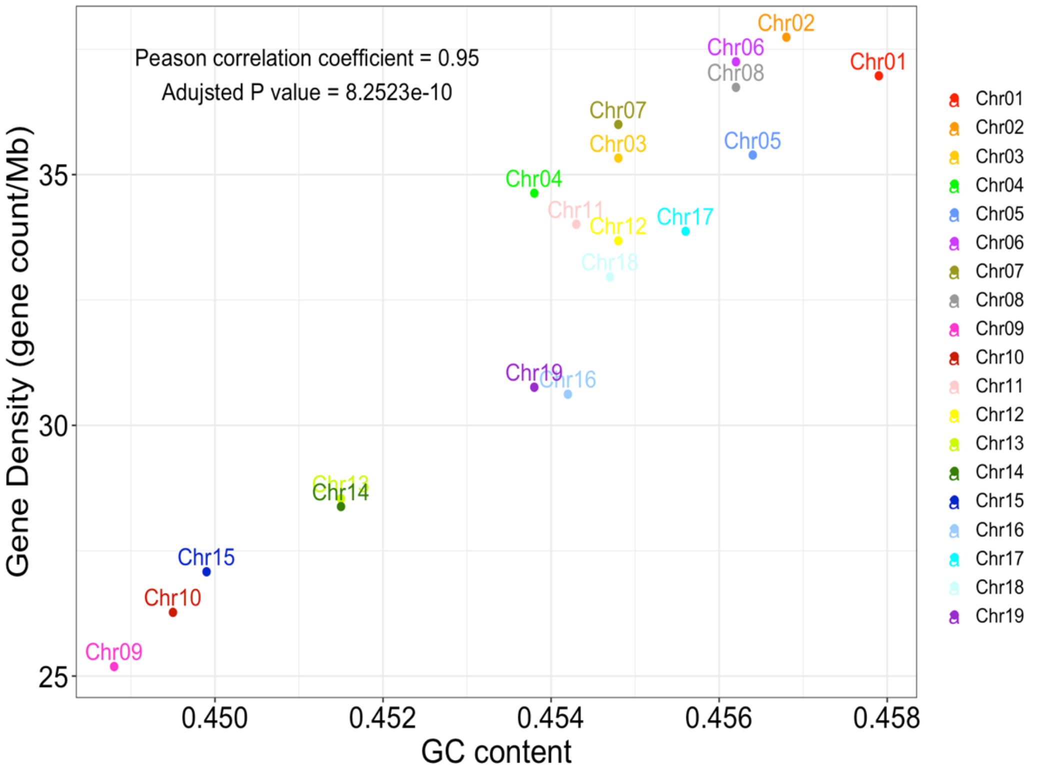

Supplementary Fig. 13. Correlation analysis of GC content and gene density. Pearson's rho rank correlation coefficient was computed using the R function rcorr within Hmisc package.

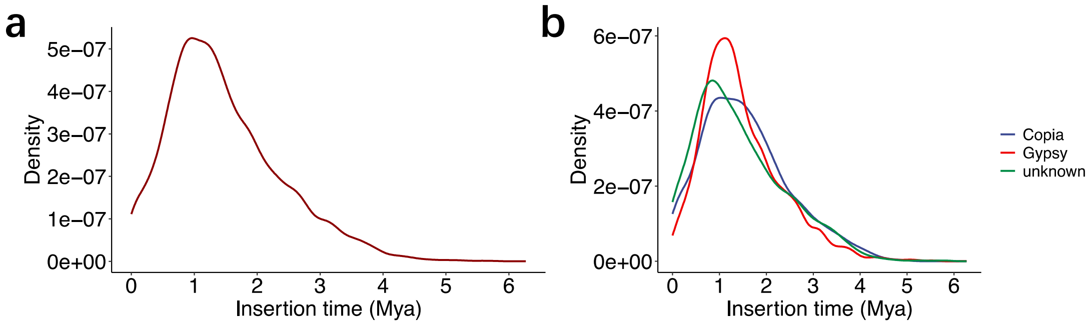

Supplementary Fig. 14. The frequency distribution of intact LTR-RTs insertion time. **a** The frequency distribution of insertion time of intact LTR-RTs in *M. lutarioriparius* genome. **b** The detailed frequency distribution of insertion time of intact *Copia*, *Gypsy* and other unclassified LTR-RTs in *M. lutarioriparius* genome.

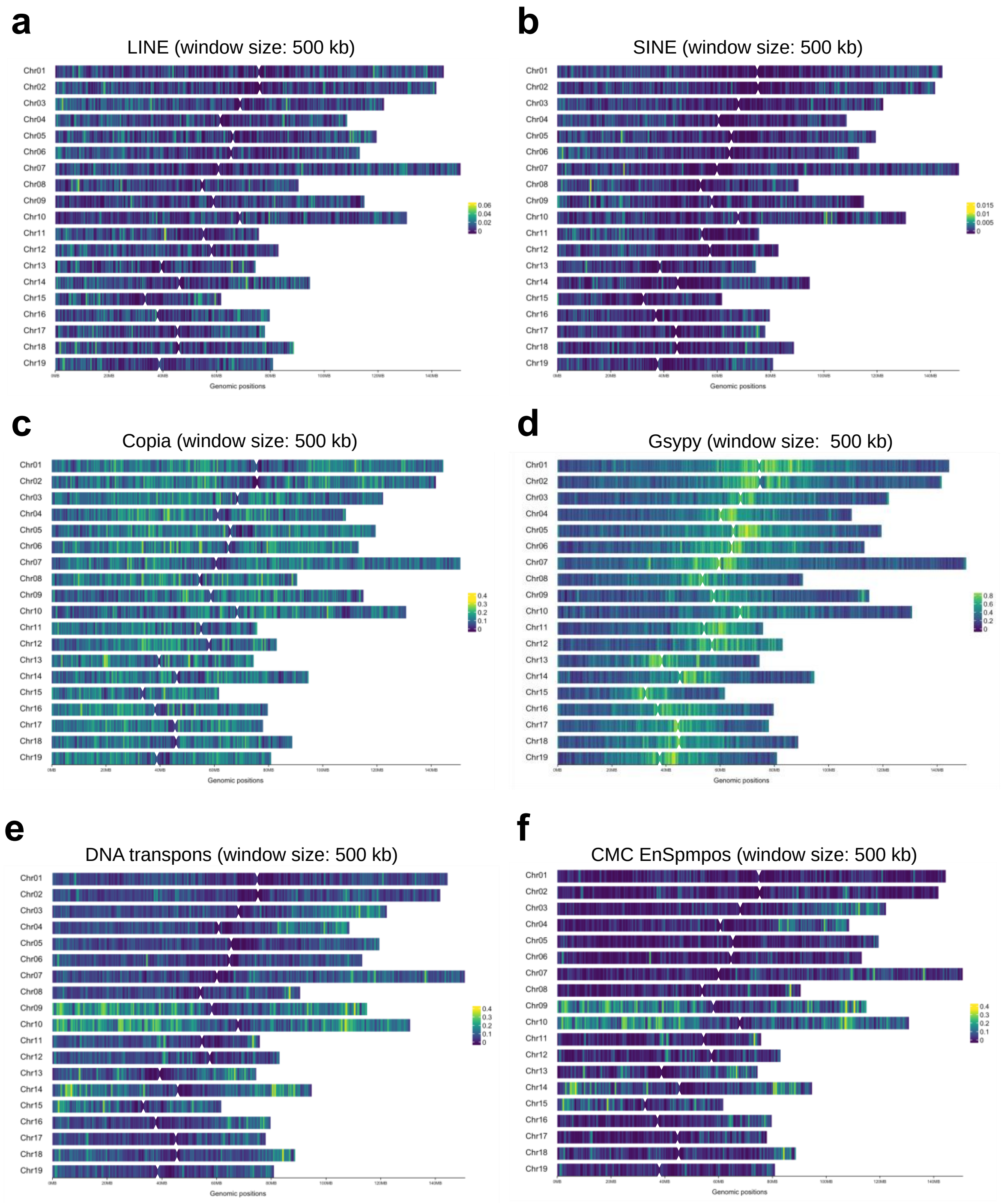

Supplementary Fig. 15. The density distribution of repeat elements across 19 chromosomes. The density for each type repeat elements is plotted using a non-overlap sliding window approach. The window size used for all repeat elements above is 500 kb. The chromosomal incisions indicate the location of centromeres. **a** LINE (Long Interspersed Nuclear Element). **b** SINE (Short Interspersed Nuclear Element). **c** *Copia*. **d** *Gspsy*. **e** DNA transposons. **f** CMC EnSpmpos.

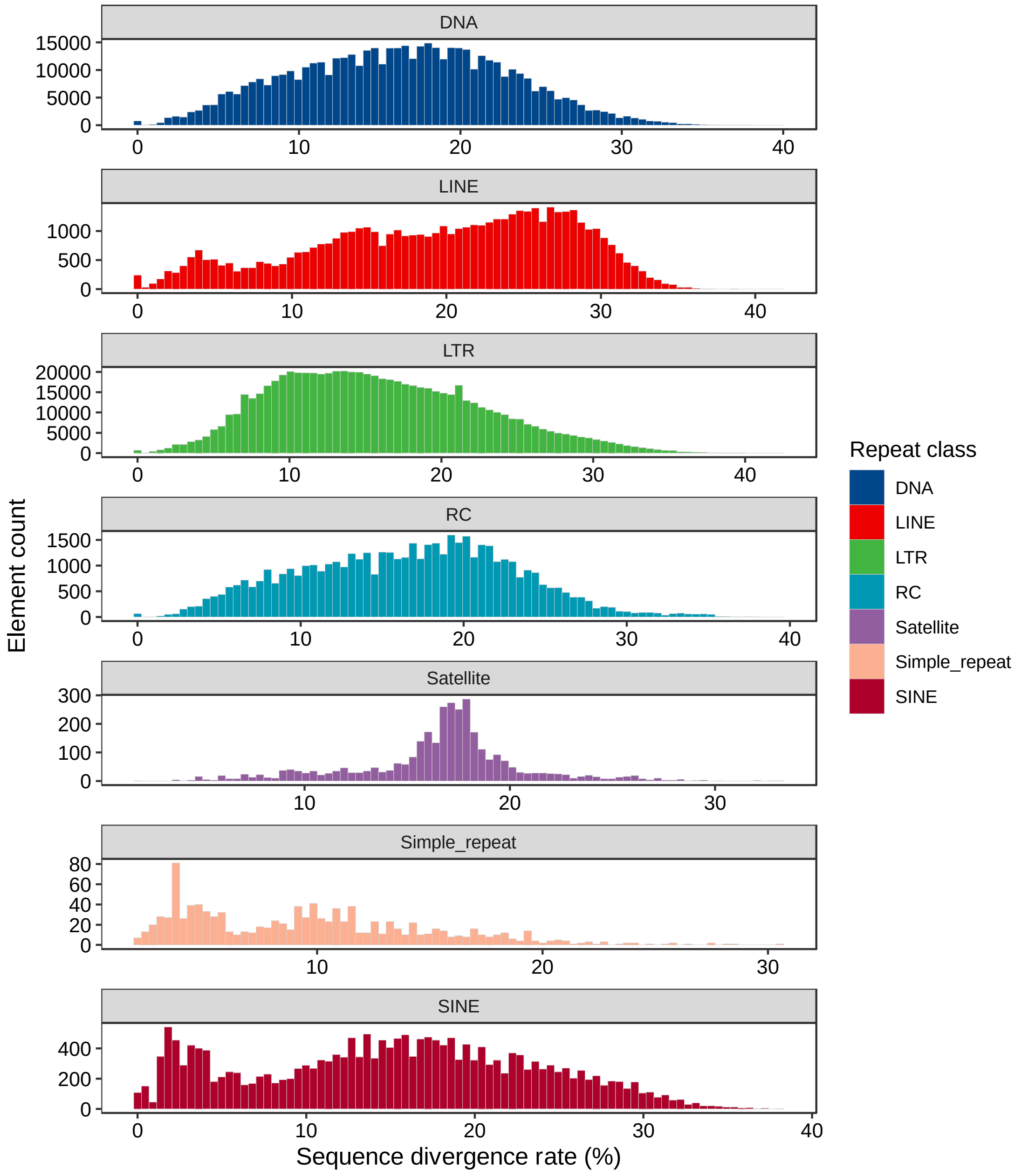

Supplementary Fig. 16. Distribution of divergence rates of different transposable element (TE) types in *M. lutarioriparius* genome. Definitions of the abbreviations follow: DNA, DNA transposon; LINE, Long Interspersed Nuclear Element; LTR, Long Terminal Repeat retroelement; RC, RC/Helitron; SINE, Short Interspersed Nuclear Element.

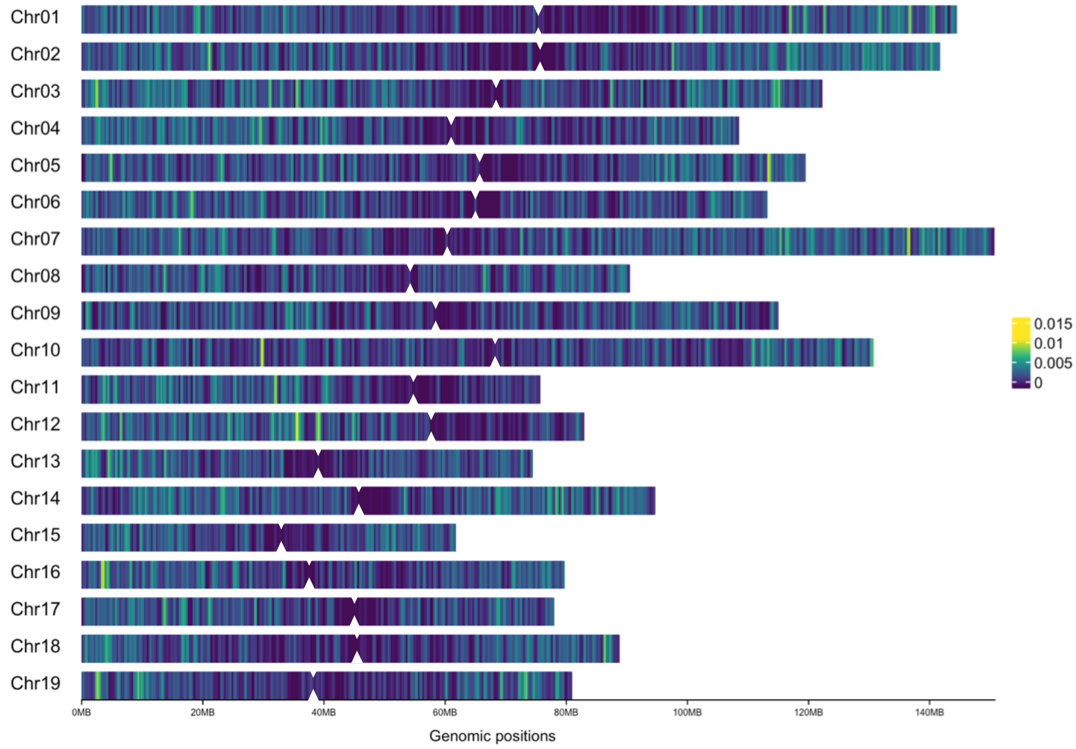

Supplementary Fig. 17. The density distribution of MITEs.

a

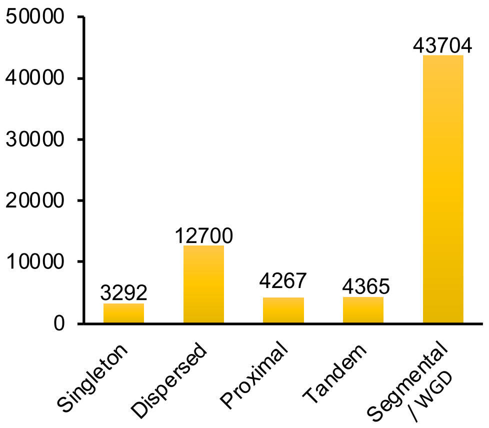

b

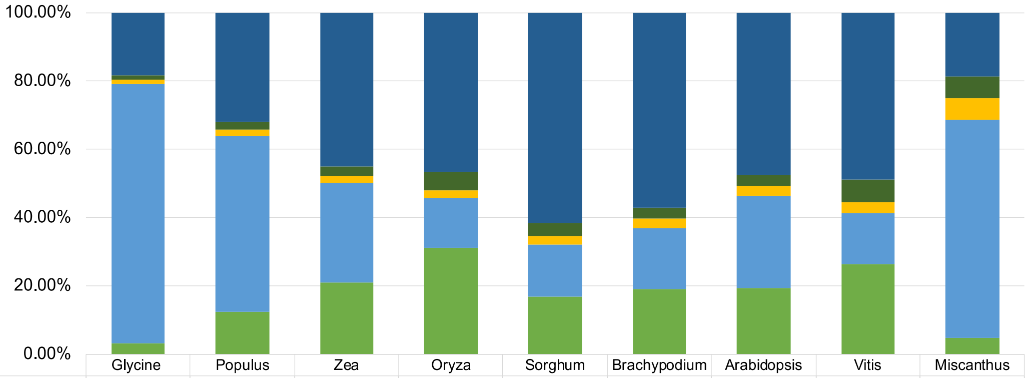

	Glycine	Populus	Zea	Oryza	Sorghum	Brachypodium	Arabidopsis	Vitis	Miscanthus
Dispersed (%)	18.15%	31.82%	44.96%	46.45%	61.59%	57.13%	47.41%	48.86%	18.59%
Proximal (%)	1.45%	2.46%	2.98%	5.37%	3.71%	3.24%	3.29%	6.72%	6.24%
Tandem (%)	1.26%	1.75%	1.97%	2.36%	2.59%	2.73%	2.84%	2.91%	6.39%
WGD (%)	76.00%	51.63%	29.23%	14.51%	15.22%	17.92%	27.01%	14.97%	63.96%
Singletons (%)	3.15%	12.33%	20.86%	31.30%	16.89%	18.97%	19.45%	26.54%	4.82%

Supplementary Fig. 18. Different origins of genes in *M. lutarioriparius* genome. a The number of genes for each duplication mode. The origin of genes in *M. lutarioriparius* were classified into singleton (no duplication), dispersed (duplication type other than WGD/segmental, tandem and proximal), proximal (two duplicated genes are distributed adjacent to each other on chromosomes, with no more than 10 genes spaced but not adjacent), tandem (consecutive repeat) and segmental/whole genome duplications (collinear genes in collinear blocks) using MCScanX[1]. **b** The numbers of genes from different origins in nine angiosperm genomes. Except *Miscanthus*, the statistics of gene origins of other eight angiosperms come from the MCScanX software paper[1].

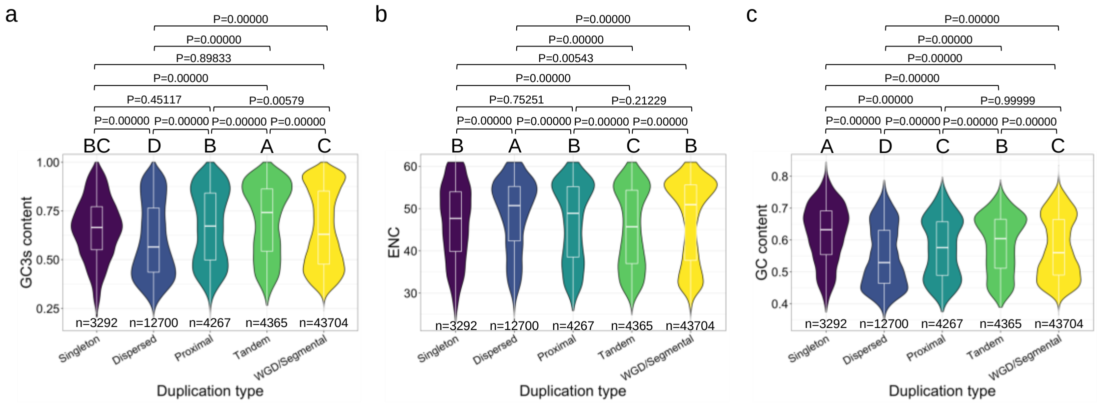

Supplementary Fig. 19. The distribution of GC3s, effective number of codons (ENC/Nc) and GC content for genes resulted from different duplication mode. a GC3s content. **b** Effective number of codons (ENC/Nc). **c** GC content. The different duplication type (Singleton, Dispersed, Proximal, Tandem and WGD/Segmental) labeled with different capital letters differ significantly in GC3s, effective number of codons and GC content (Statistical comparison was carried out using ANVOA followed by Tukey Honest Significant Differences analysis, $P < 0.01$). In each boxplot, the center line indicates the median, the lower and upper hinges represent the first and third quartiles, the upper whisker extends to the largest value less than 1.5× the interquartile range (IQR), the lower whisker extends to the smallest value at most 1.5× the IQR, the black point represent outlier. The number of data points used for plotting is shown at the bottom.

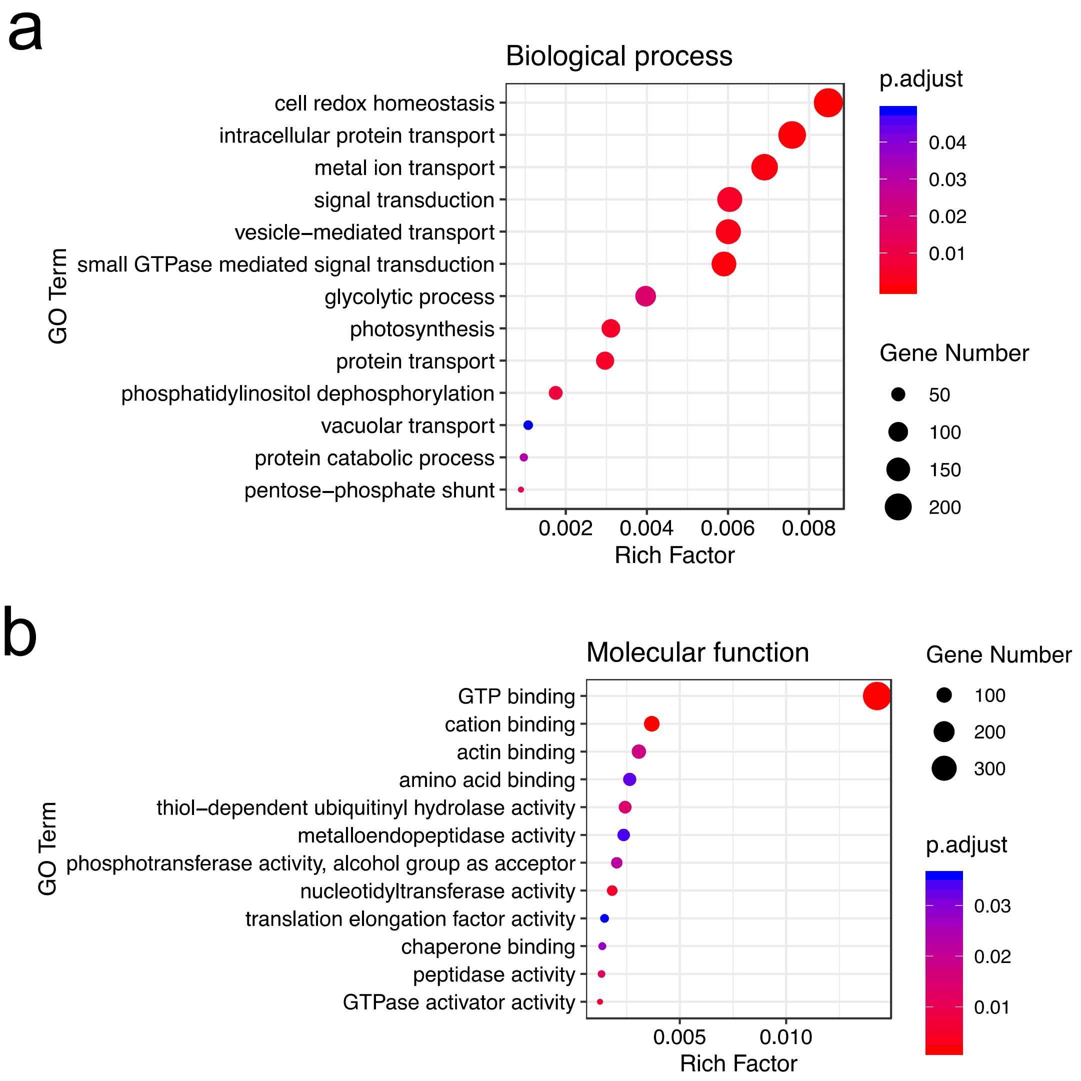

Supplementary Fig. 20. Gene Ontology (GO) enrichment analysis of *M. lutarioriparius* WGD/segmental duplication. a GO enrichment results of biological process category. The color of circle represents the FDR (false discovery rate) in the hypergeometric test corrected using BH method[2]. The size of circle represents the gene count of the GO terms. The rich factor is defined as gene count / total query gene count. **b** GO enrichment results of molecular function category. The color of circle represents the FDR (false discovery rate) in the hypergeometric test corrected using BH method[2]. The size of circle represents the gene count of the GO terms. The rich factor is defined as gene count / total query gene count.

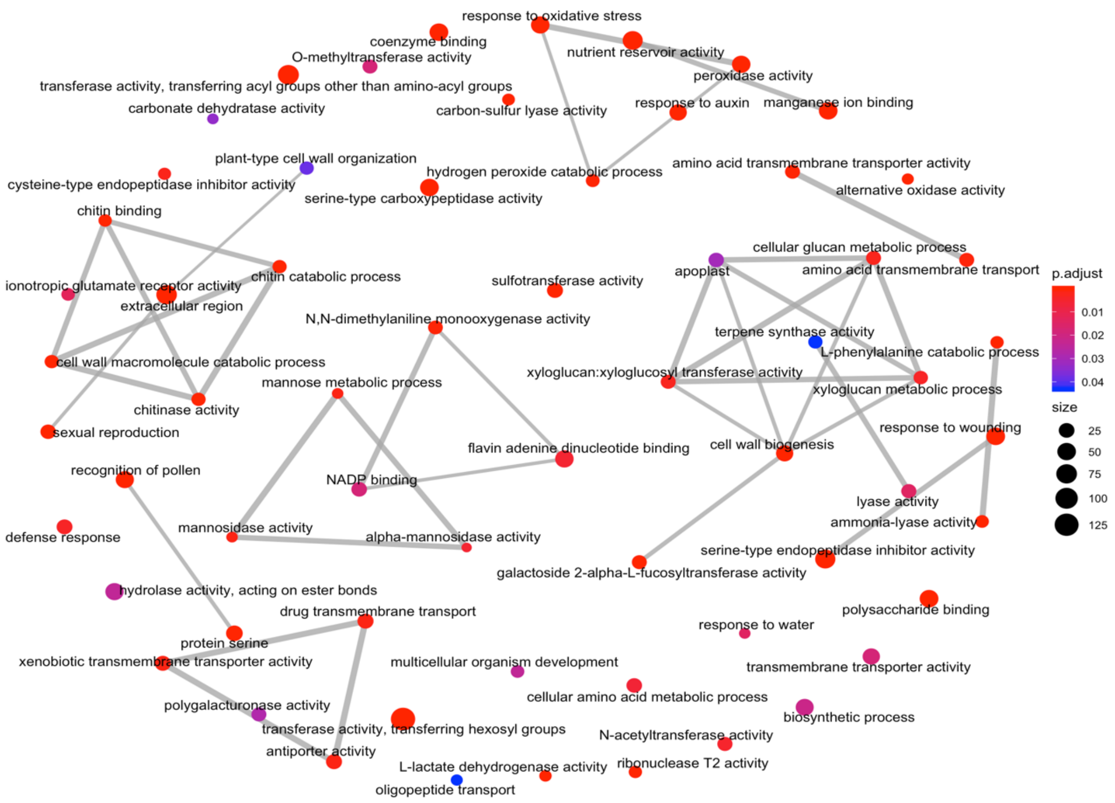

Supplementary Fig. 21. Enrichment Map for enrichment result of over-representation test for gene duplicates from tandem duplication. Mutually overlapping gene sets tend to cluster together, making it easier for interpretation.

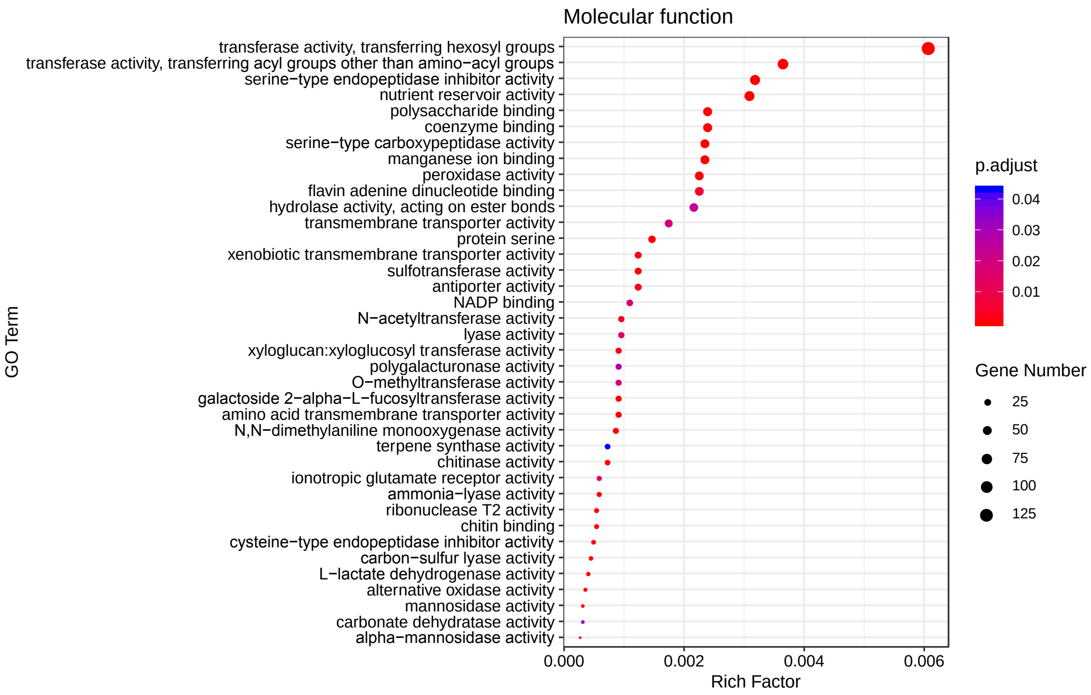

Supplementary Fig. 22. Gene Ontology (GO) enrichment analysis of *M. lutarioriparius* genes resulted from tandem duplication (molecular function category). The color of circle represents the FDR (false discovery rate) in the hypergeometric test corrected using BH method[2]. The size of circle represents the gene count of the GO terms. The rich factor is defined as gene count / total query gene count.

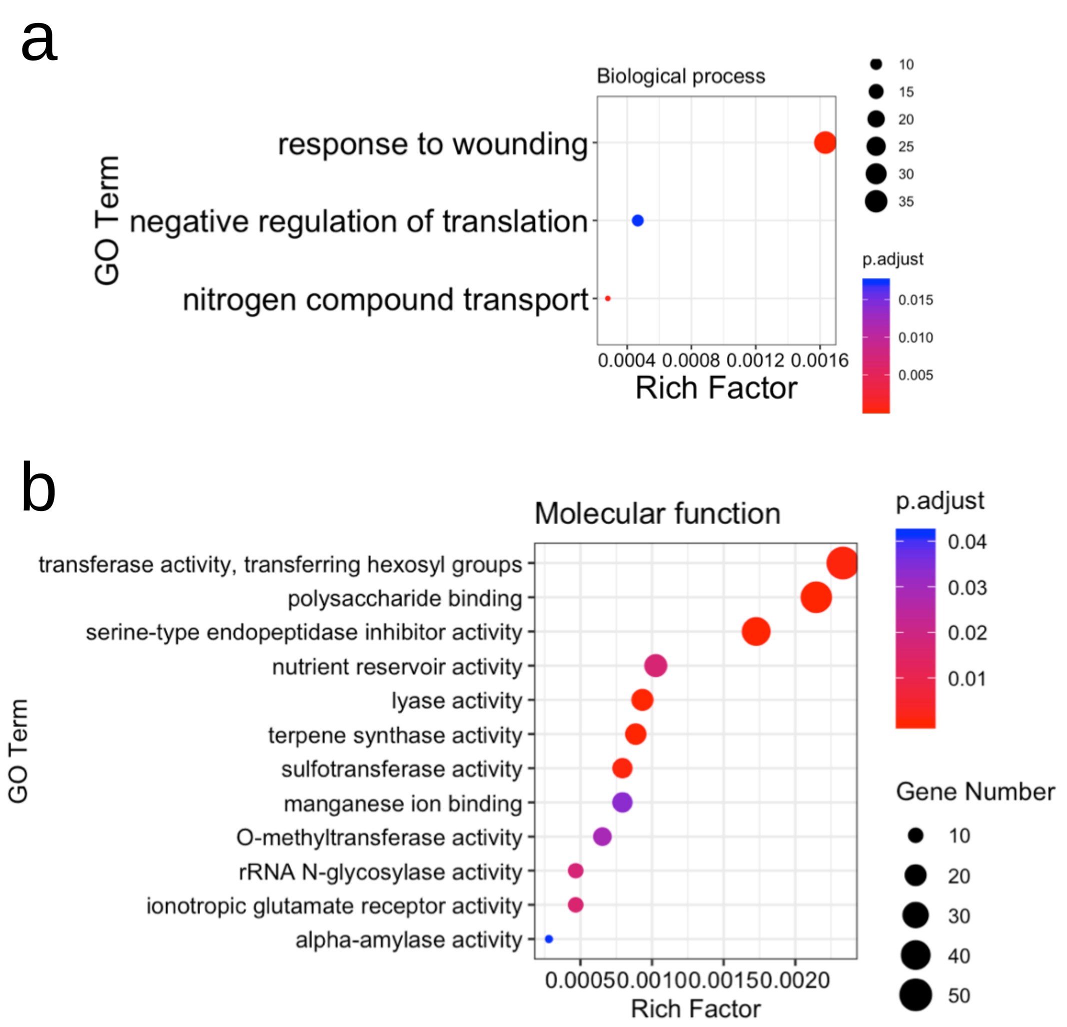

Supplementary Fig. 23. Gene Ontology (GO) enrichment analysis of *M. lutarioriparius* gene duplicates from proximal duplication. a Biological process category. The color of circle represents the FDR (false discovery rate) in the hypergeometric test corrected using BH method[2]. The size of circle represents the gene count of the GO terms. The rich factor is defined as gene count / total query gene count. **b** Molecular function category. The color of circle represents the FDR (false discovery rate) in the hypergeometric test corrected using BH method[2]. The size of circle represents the gene count of the GO terms. The rich factor is defined as gene count / total query gene count.

a

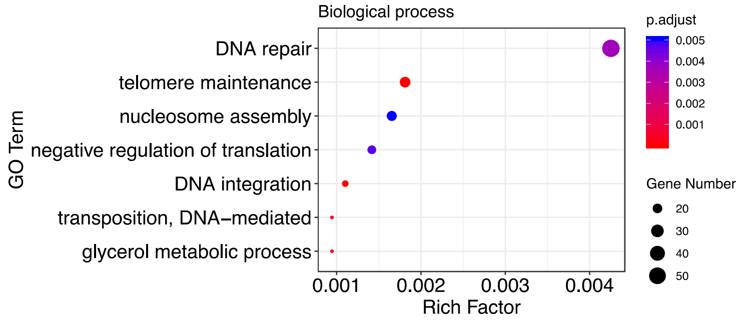

b

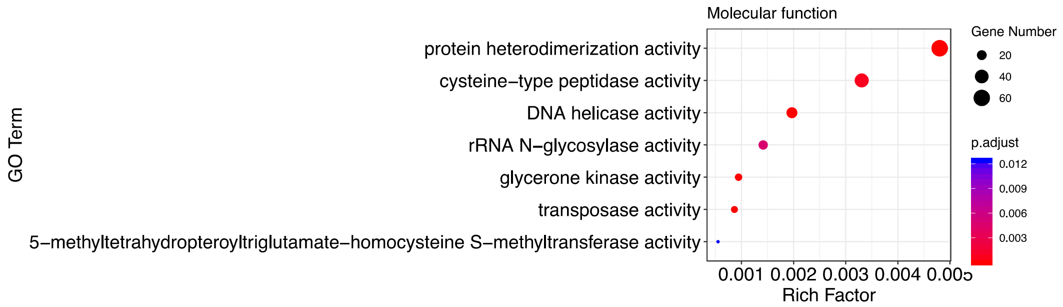

Supplementary Fig. 24. Gene Ontology (GO) enrichment analysis of *M. lutarioriparius* gene duplicates of dispersed duplication origin. a GO enrichment results of biological process category. The color of circle represents the FDR (false discovery rate) in the hypergeometric test corrected using BH method[2]. The size of circle represents the gene count of the GO terms. The rich factor is defined as gene count / total query gene count. **b** GO enrichment results of molecular function category. The color of circle represents the FDR (false discovery rate) in the hypergeometric test corrected using BH method[2]. The size of circle represents the gene count of the GO terms. The rich factor is defined as gene count / total query gene count.

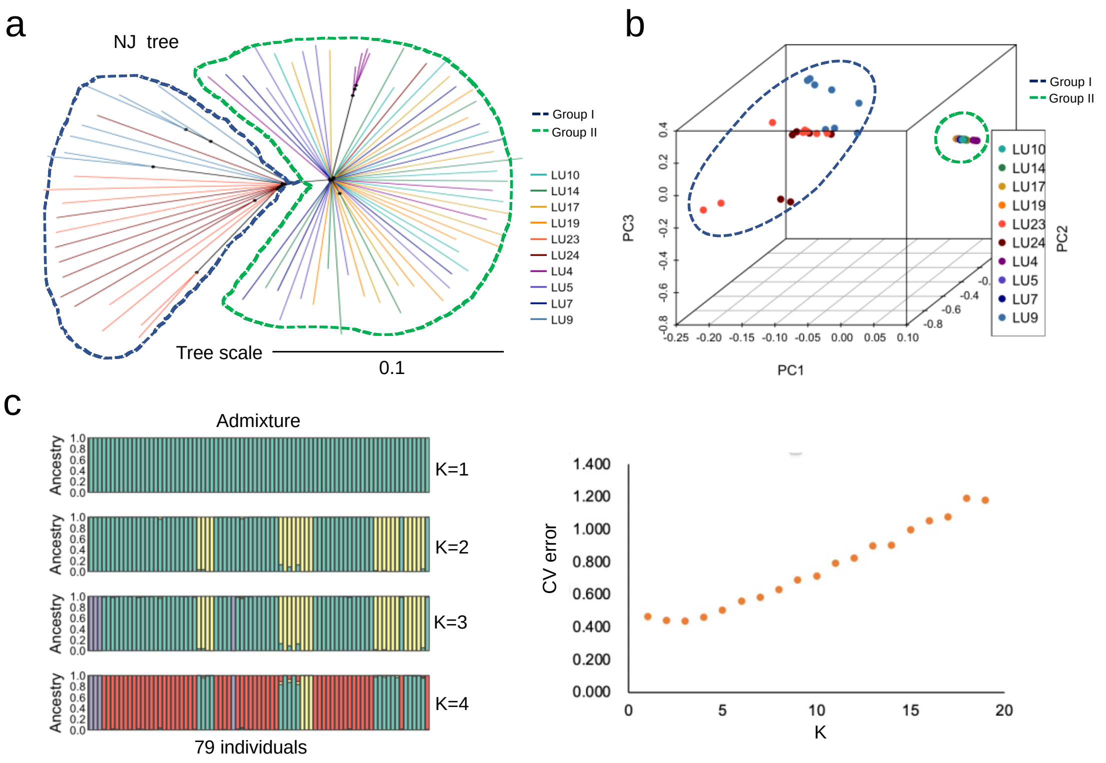

Supplementary Fig. 25. Genetic diversity analysis of *Miscanthus lutarioriparius* populations based on transcriptome data. a Neighbor-joining tree reconstructed using the SNPs identified by transcriptome data. Lines of different colors indicate different populations of *Miscanthus lutarioriparius*. The dotted lines in green and blue represent Group I and Group II, respectively. **b** Principal component analysis based on the SNPs identified by transcriptome data. Points of different colors indicate different populations of *Miscanthus lutarioriparius*. Two circles indicate Group I and II of *Miscanthus lutarioriparius*. Our sequenced *M. lutarioripairus* is belong to Group II (not show in this plot). **c** Admixture analysis based on the SNPs identified by transcriptome data.

a

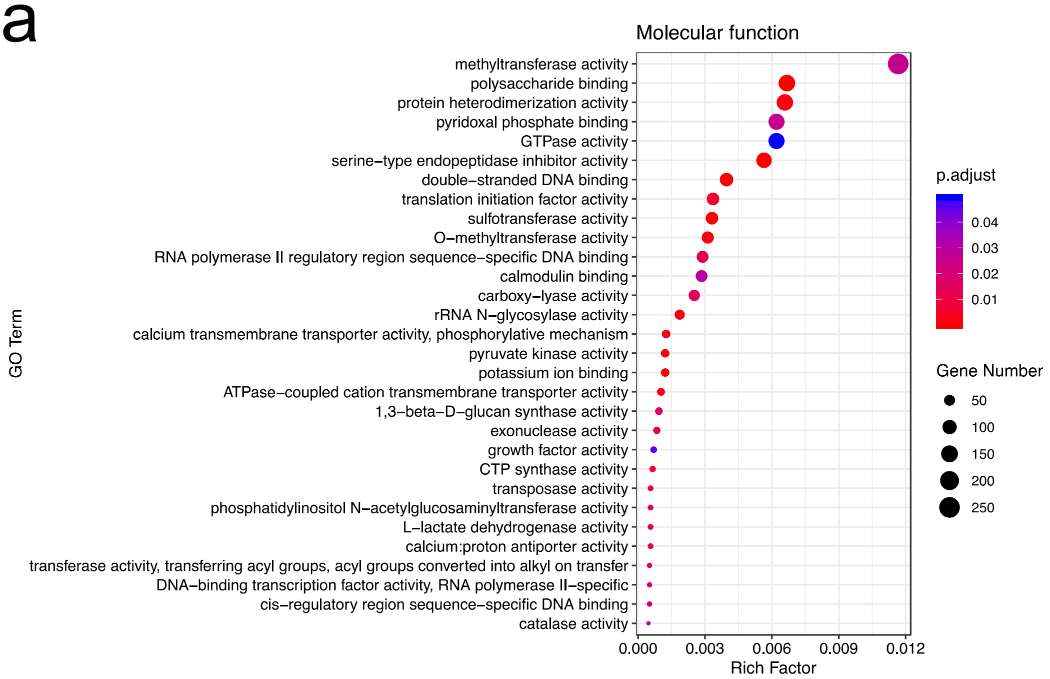

b

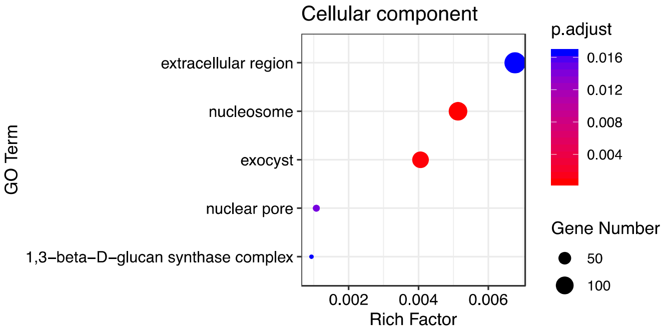

Supplementary Fig. 26. Dot-plot of GO enrichment for 9,509 expanded gene families of *M. lutarioriparius*. a GO enrichment results of biological process category. The color of circle represents the FDR (false discovery rate) in the hypergeometric test corrected using BH method[2]. The size of circle represents the gene count of the GO terms. The rich factor is defined as gene count/total query gene count. **b** GO enrichment results of cellular component category. The color of circle represents the FDR (false discovery rate) in the hypergeometric test corrected using BH method[2]. The size of circle represents the gene count of the GO terms. The rich factor is defined as gene count/total query gene count.

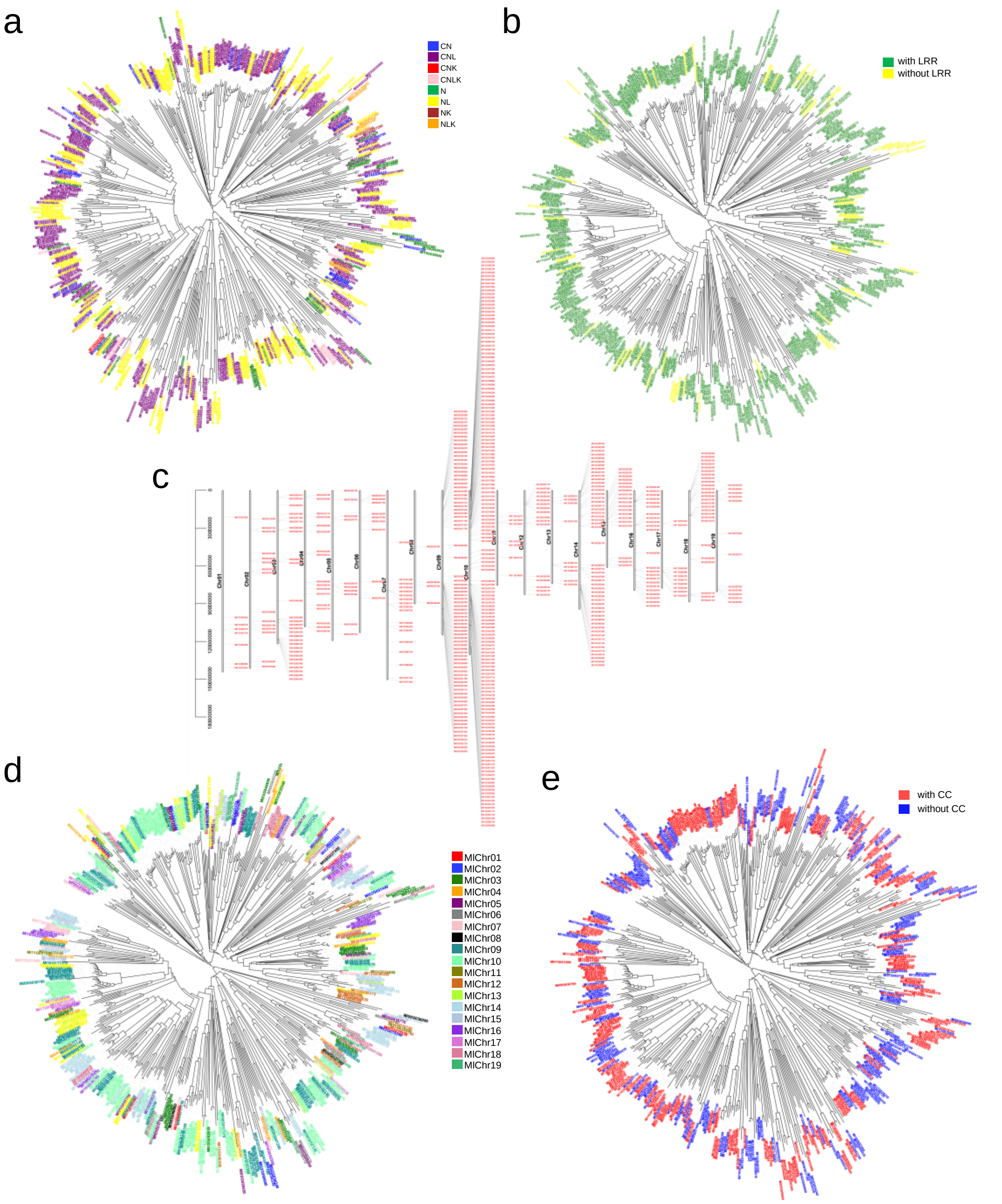

Supplementary Fig. 27. Phylogenetic tree reconstructed based on the amino acid sequences in the NBS domain of NBS-encoding genes from *M. lutarioriparius.* a Color-coded by classification of NBS-encoding genes. **b** Color-coded by presence or absence of LRR domain. **c** Genomic distribution of NBS-encoding genes. **d** Color-coded by presence or absence of CC domain. **e** Color-coded by chromosome as indicated.

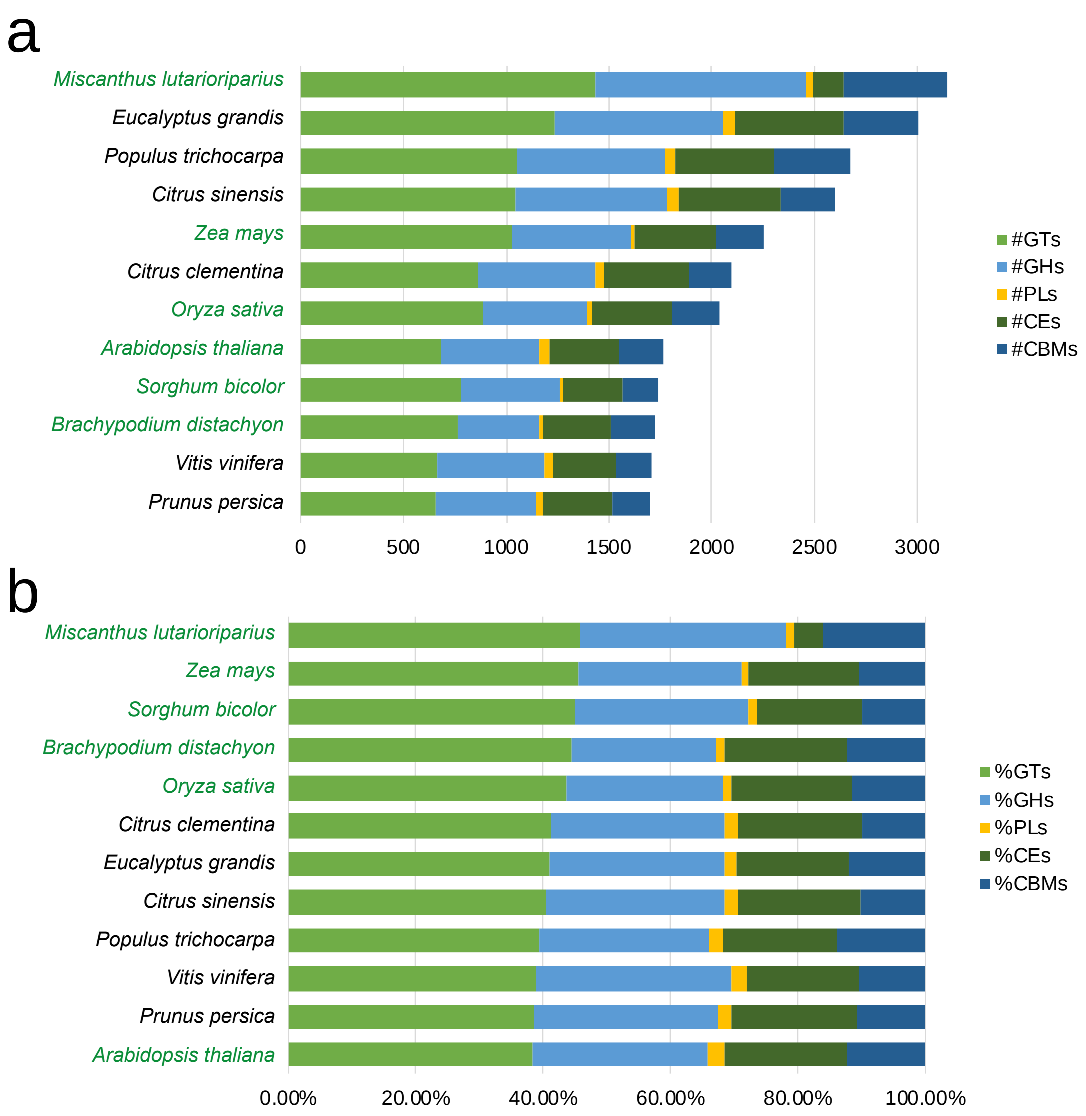

Supplementary Fig. 28. CAZyme domain classes frequency distribution in twelve plant species. a Absolute frequency of CAZyme domain classes in twelve plant species. Plant species are on the y-axis, and the frequency of CAZyme domains within all CAZyme genes is shown on the x-axis. The green color of plant species is used to indicate the herbaceous species, while the black is used to indicate the woody species. The glycosyl transferase (GT) domain class is indicated using green, glycosyl hydrolase (GH) domain class using blue, polysaccharide lyase (PL) domain class using yellow, carbohydrate esterase (CE) domain class using dark green and carbohydrate binding module (CBM) domain class using dark blue. Except *Miscanthus lutarioriparius*, the other data came from Pinard et al.,[3]. **b** Relative frequency of CAZyme domain classes in twelve plant species. The relative frequency of carbohydrate active enzyme (CAZyme) domain classes, as a percentage, is shown on the x-axis. The species of plant is shown on the y-axis.

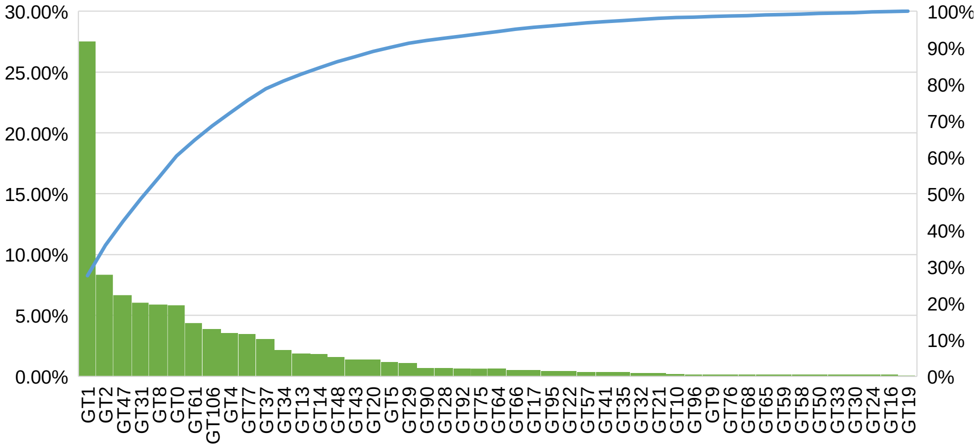

Supplementary Fig. 29. Frequency distribution of gene members of GT family.

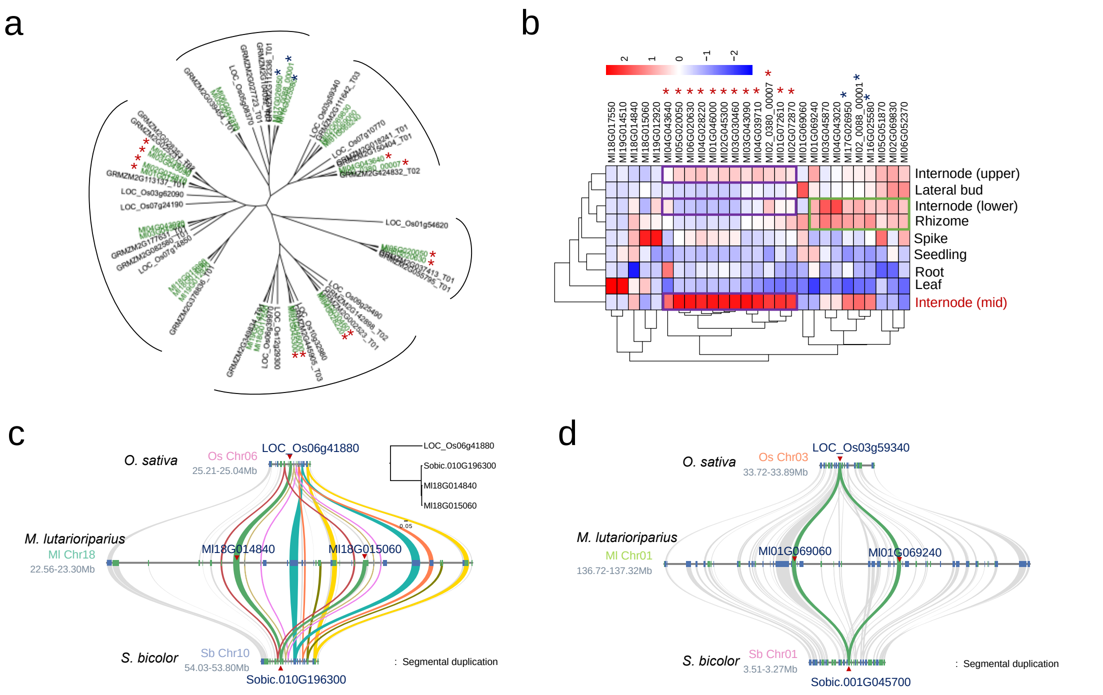

Supplementary Fig. 30. *CesA* genes of *M. lutarioriparius*. a Phylogeny tree of *CesA* genes reconstructed using maximum likelihood approach by running IQtree software using protein sequences of *M. lutarioriparius*, rice and maize. Green color is used to indicate the genes of *M. lutarioriparius.* **b** Expression pattern of *CesA* genes in 9 transcriptome samples. Red and blue stars were used to indicate the *CesA* genes that have specifically high expression in the mid of internode. **c** Segmental duplication leads to the expansion of *M. lutarioriparius CesA* genes (M18G014840 and Ml18G015060). **d** Segmental duplication leads to the expansion of *M. lutarioriparius CesA* genes (M01G069060 and Ml01G069240).

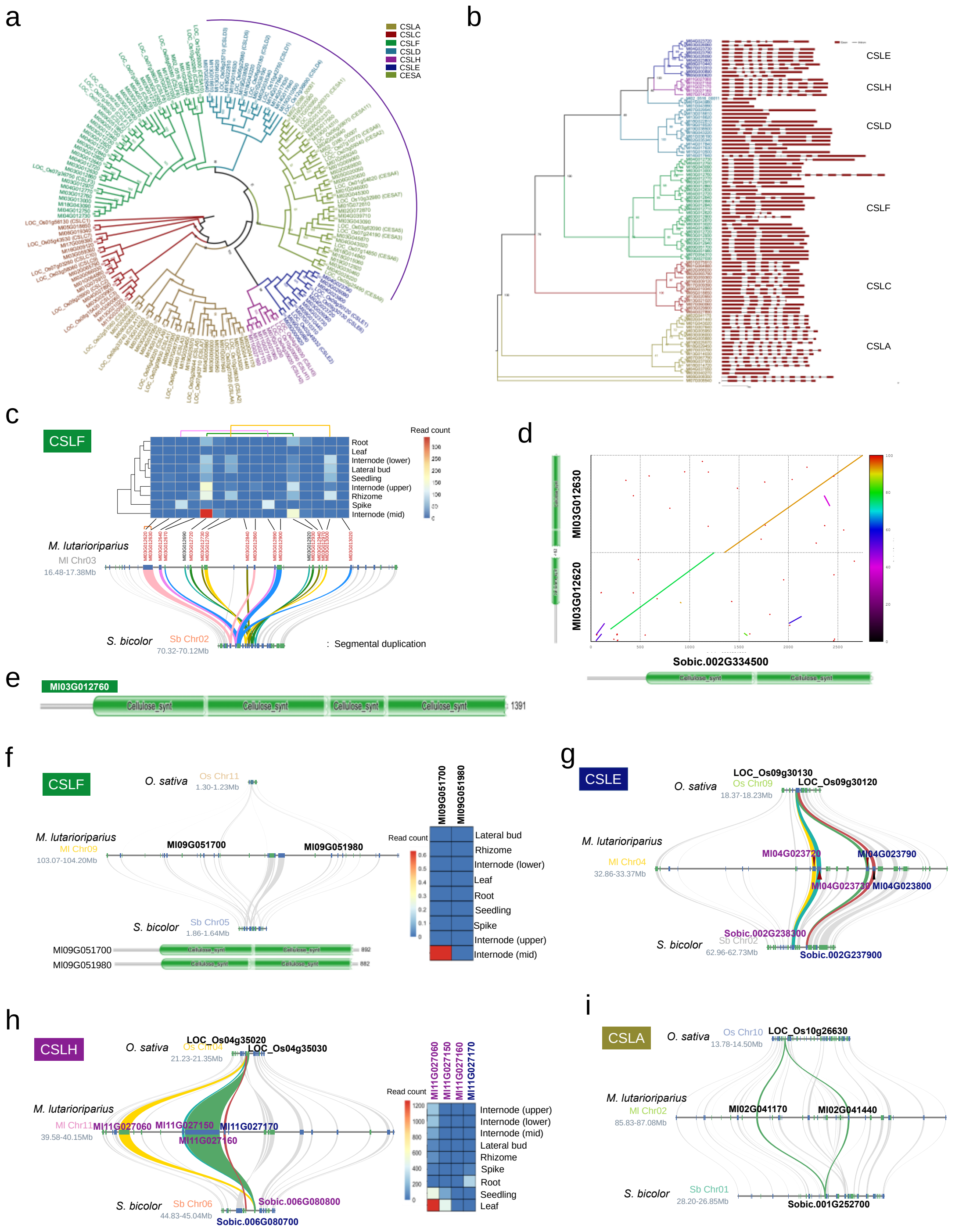

Supplementary Fig. 31. Csl gene family of *M. lutarioriparius.* a Phylogeny tree of Csl and CesA of *M. lutarioripaiurs* was reconstructed using maximum likelihood approach. Different colors were used to indicate the groups. Bootstrap values (integer) are indicated at the nodes. **b** Gene structure diagram of Csl gene family of *M. lutarioriparius*. Red rectangles and black lines were used to indicate the exons and introns, respectively. **c** The segmental duplication of CslF genes was occurred in *M. lutarioriparius* after it's split with sorghum. And expression heatmap of CslF gene family in 9 transcriptome samples. Links with same color is used to indicate the syntenic gene pairs that share common sorghum genes. The red gene symbols of *M. lutarioriparius* are Csl genes. The expression pattern of CslF genes in 9 transcriptome samples are shown using heatmap. **d** Comparison of sequence similarity between Ml03G012620 and Ml03G012630. **e** Gene fusion occurred in Ml03G012760. Pfam domain of Cellulose_synt (PF03552) is indicated by green rectangle.

f *M. lutarioriparius* specific duplication of CslF genes, which have no homolog in the expected collinear regions in both sorghum and rice genomes. These two Csl genes were confirmed by Pfam annotation. Heatmap is used to show the library-size normalized read count of Ml09G05170 and Ml09G051980, showing very low expression for both genes in 9 transcriptome samples. **g** Tandem duplication of CslE genes occurred in *M. lutarioriparius* after its split with sorghum. **h** Tandem and proximal duplication of CslH genes occurred in *M. lutarioriparius* after its split with sorghum. **i** Proximal duplication of CslA genes in *M. lutarioriparius* after its split with sorghum. The green lines were used to indicate the CslA genes collinearity between *M. lutarioriparius* and sorghum.

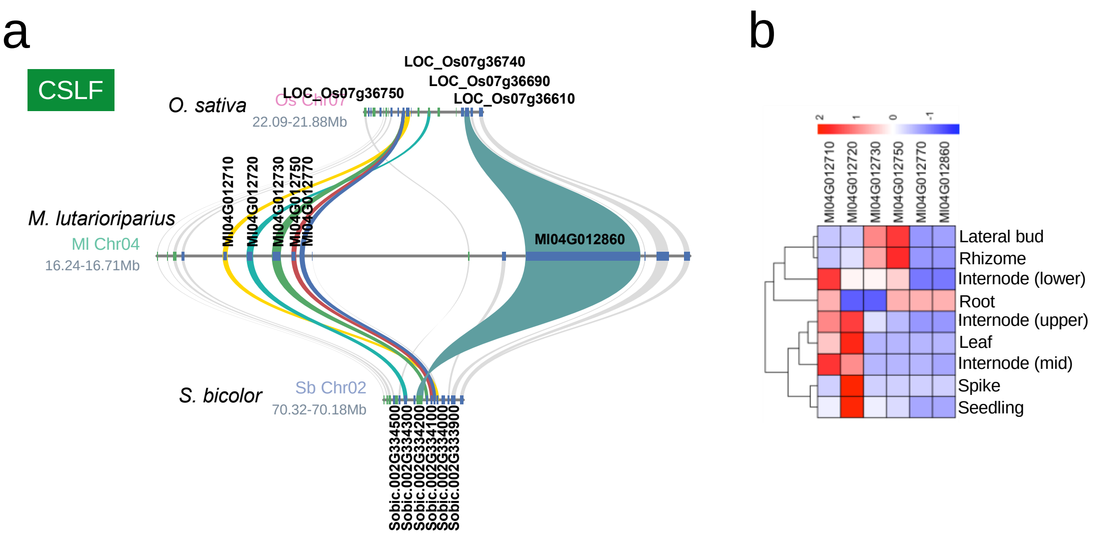

Supplementary Fig. 32. The marcosynteny of CslF family among *M. lutarioriparius*, rice and sorghum. **a** The syntenic gene pairs between *M. lutarioriparius*, rice and sorghum. **b** The expression pattern of gene members in CslF family of *M. lutarioriparius*.

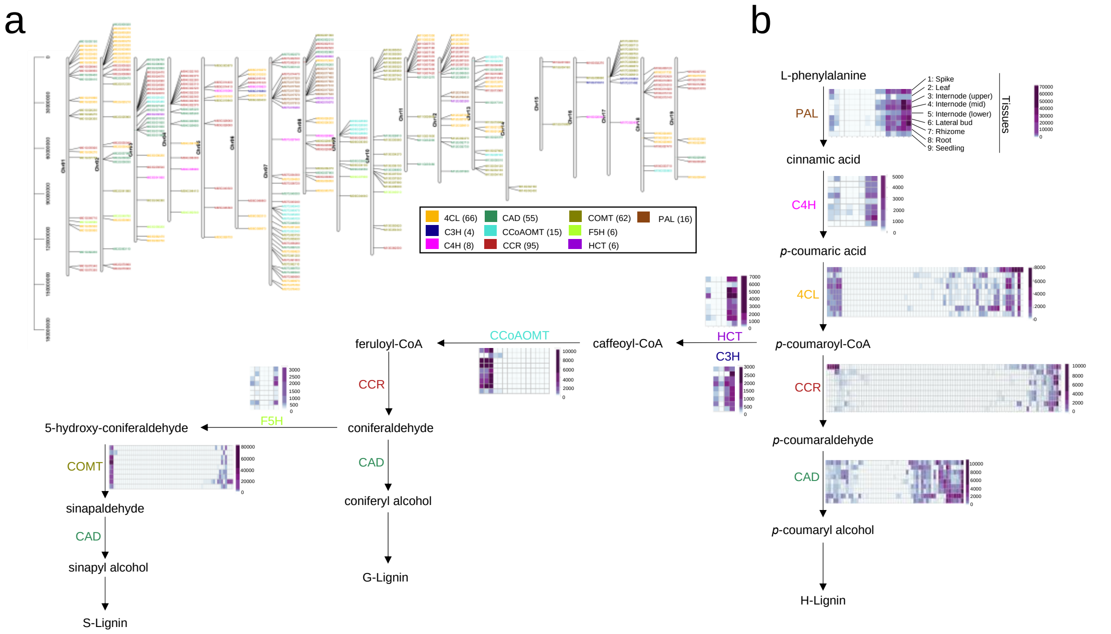

Supplementary Fig. 33. Genes involved in lignin biosynthesis in *M. lutarioriparius.* a Gene position on chromosomes. Legend: the numbers in parentheses indicate gene counts. **b** Gene expression heatmap (normalized read count) and lignin biosynthesis pathway. 4CL: 4-coumarate: CoA ligase; C3H: p-coumarate-3-hydroxylase; C4H: Trans-cinnamate 4-hydroxylase; CAD: cinnamyl alcohol dehydrogenase; CCoAOMT: Caffeoyl coenzyme A-3-O-methyltransferase; CCR: cinnamoyl CoA reductase; COMT: caffeic acid O-methyltransferase; F5H: ferulate-5-hydroxylase; HCT: hydroxycinnamoyl-CoA shikimate/quinate hydroxycinnamoyl transferase; PAL: phenylalanine ammonia-lyase.

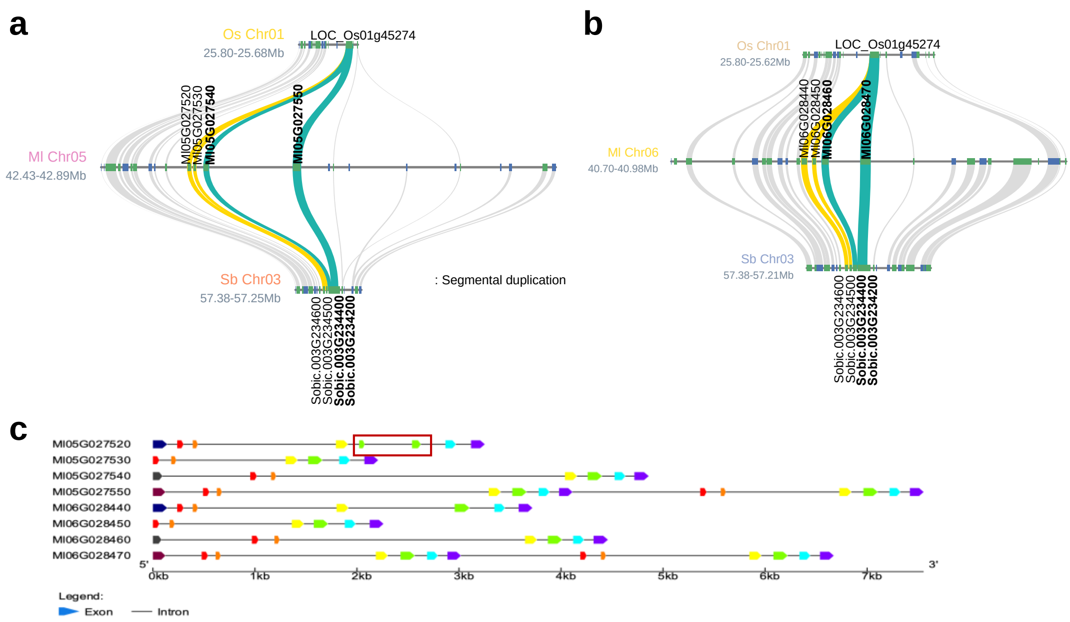

Supplementary Fig. 34. CA enzyme genes of *M. lutarioripairus* were located in two tandem duplication blocks. a Gene synteny of CA enzyme genes among *M. lutarioriparius* (chromosome 5), sorghum and rice. **b** Gene synteny of CA enzyme genes among *M. lutarioriparius* (chromosome 6), sorghum and rice. **c**, Gene structure diagram of *M. lutarioriparius* CA enzyme genes located in chromosome 5 and 6. Colored squares and black lines were used to indicate the exons and introns, respectively. Same color indicates the sequence with high similarity. Based on gene structure and sequence identity, Ml05G027550 is likely to be derived from the fusion of two neighboring genes.

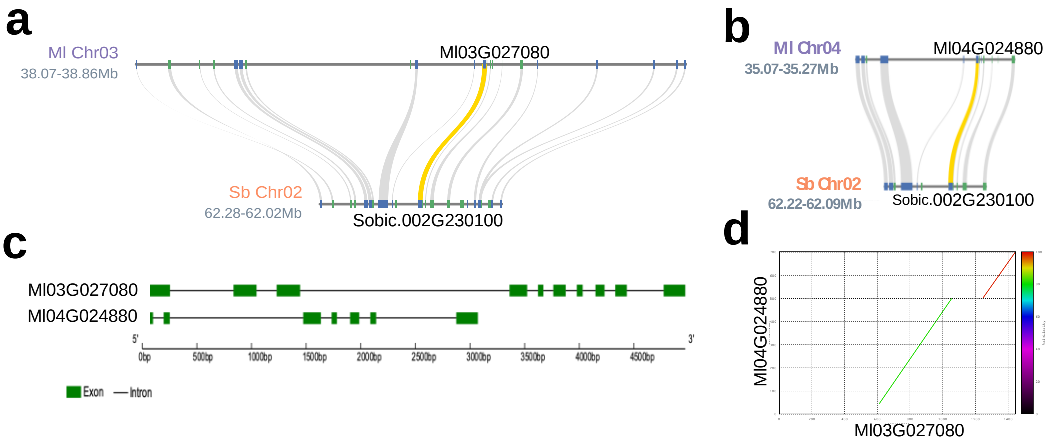

Supplementary Fig. 35. Identification of *M. lutarioriparius* CA enzyme genes Ml03G027080 and Ml04G024880. a *M. lutarioriparius* CA enzyme gene Ml03G027080 is syntenic with sorghum Sobic.002G230100. **b** *M. lutarioriparius* CA enzyme gene Ml04G024880 is syntenic with sorghum Sobic.002G230100. **c** Gene structure of CA enzyme genes Ml03G027080 and Ml04G024880. The green rectangles and black lines represent exons and introns, respectively. **d** Comparison of coding sequence between Ml03G027080 and Ml04G024880.

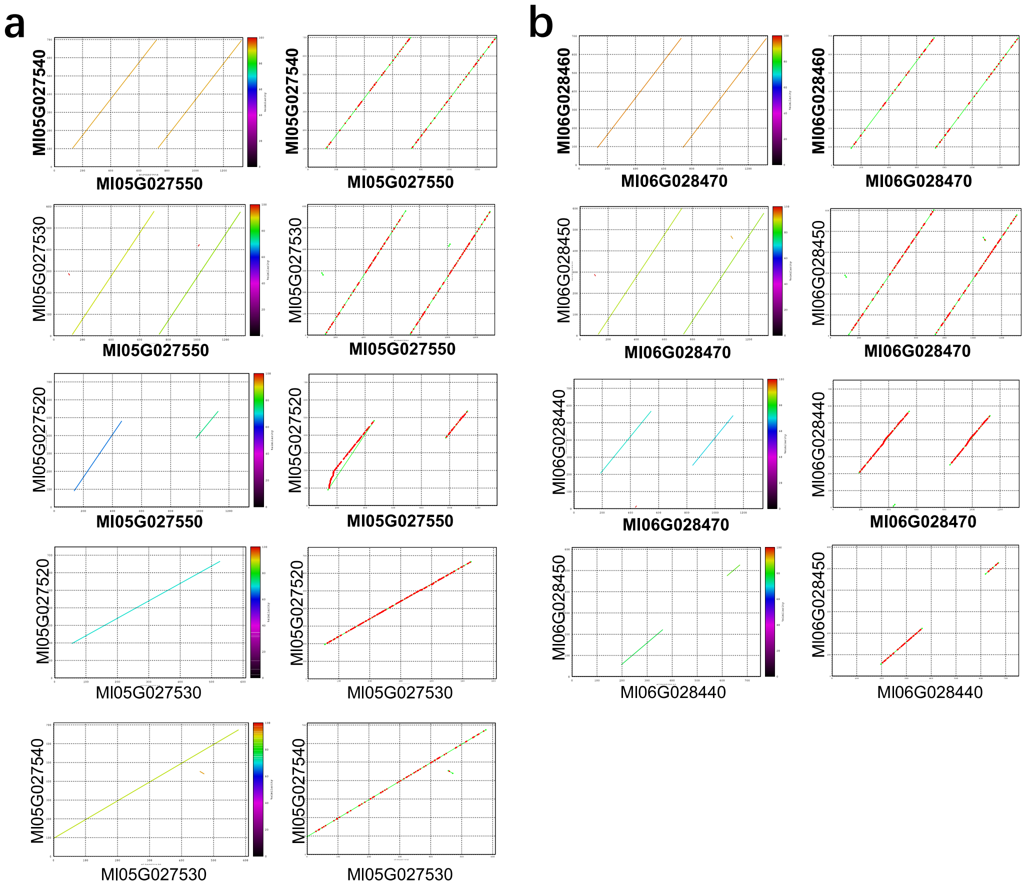

Supplementary Fig. 36. Dot-plots of *M. lutarioriparius* CA enzyme genes (CDS). a Dot-plots of *M. lutarioriparius* CA enzyme genes that located in chromosome 5, the left panel displays the overall sequence similarity between two comparing sequences, the right panel shows the SNPs with red dot between two sequences. Bold characters represent putative C_4 genes. **b** Dot-plots of *M. lutarioriparius* CA enzyme genes that located in chromosome 6.

a

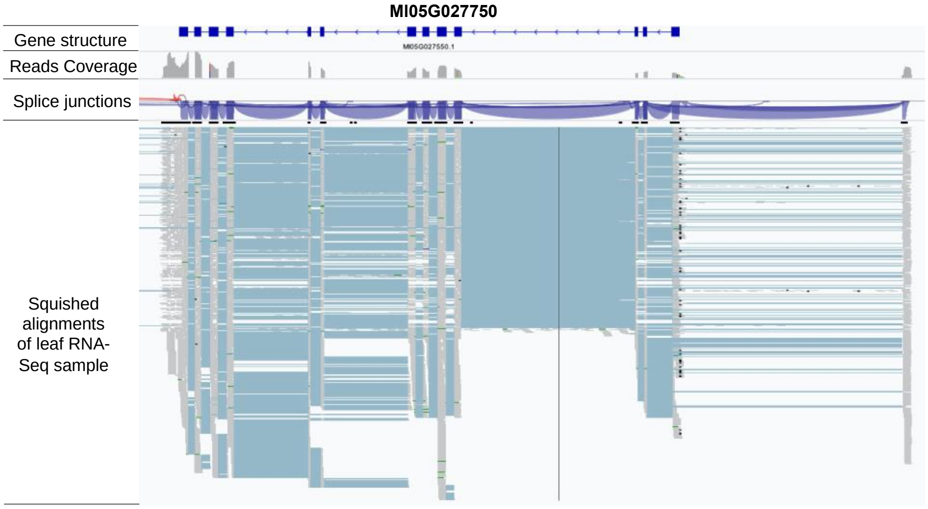

b

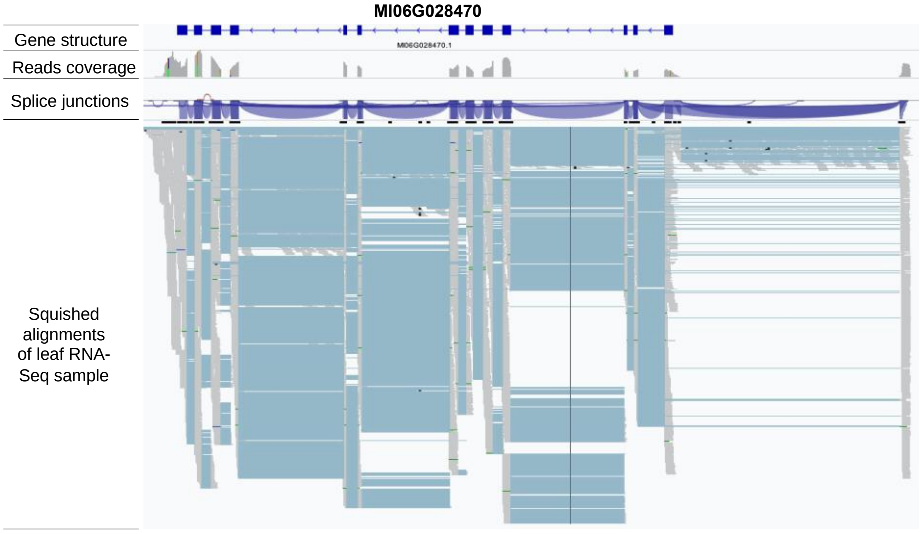

Supplementary Fig. 37. The visualization of RNA-Seq reads alignment to two CA genes (MI05G027750 and MI06G028470). a The RNA-Seq reads alignment of leaf sample on MI05G027750. The first track showed the gene structure diagram. Blue-colored rectangles and lines were used to represent exons and introns, respectively. The second track showed the leaf RNA-Seq reads coverage on gene MI05G027750. The third track showed the splice-junctions of leaf RNA-Seq reads that mapped to gene MI05G027750. The fourth track showed the squished alignments of leaf RNA-Seq reads on gene MI05G027750. The grey lines were used to indicate the RNA-Seq short reads and the light blue lines represented the splice-junctions of RNA-Seq short reads. **b** The RNA-Seq reads alignment of leaf sample on MI06G028470.

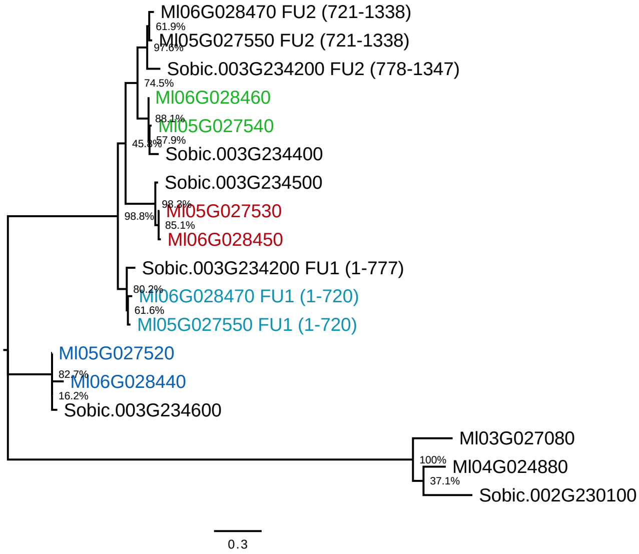

Supplementary Fig. 38. Neighbor-joining tree of *M. lutarioriparius* CA enzyme genes (CDS) and sorghum CA enzyme genes (CDS) reconstructed using MEGA X software with 1,000 replicates of bootstrap test. Numbers at nodes indicate the percentage bootstrap scores from 1,000 replicates.

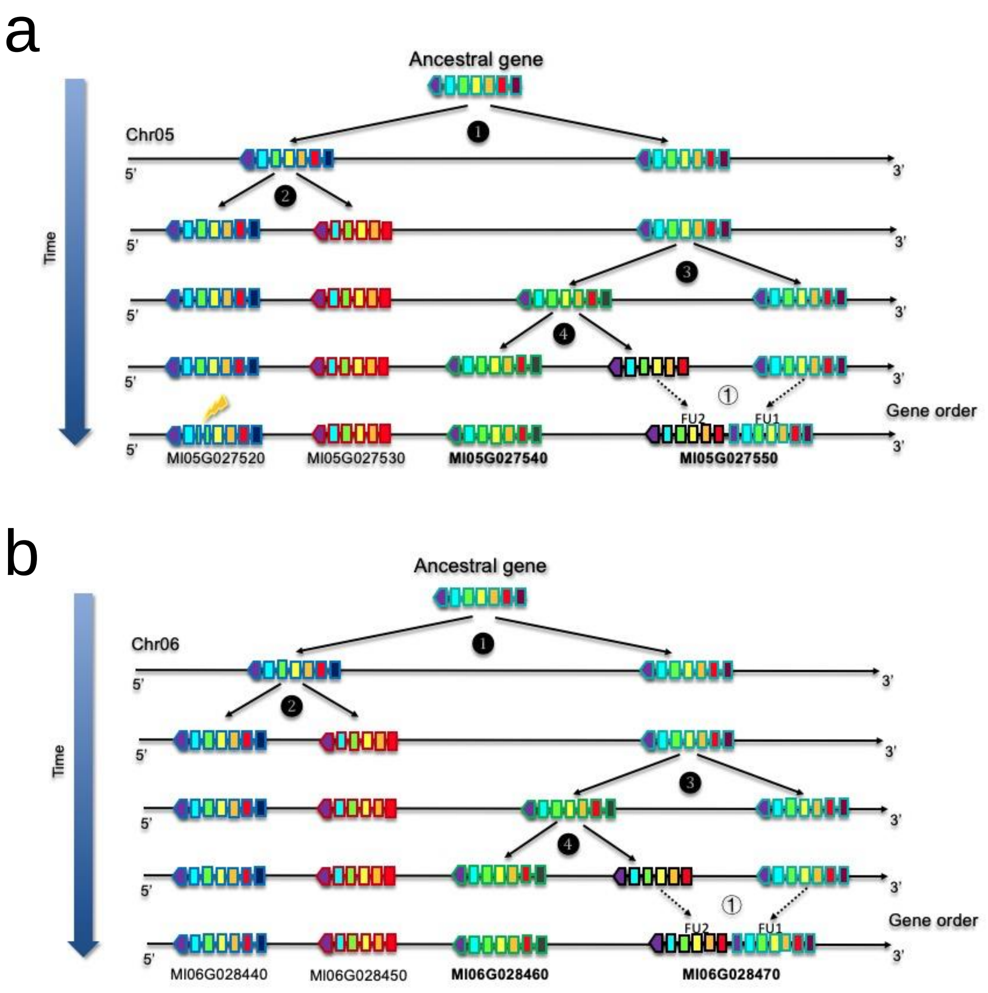

Supplementary Fig. 39. Inferred duplication evolutional model of CA enzyme genes. The CA enzyme genes in *M. lutarioriparius* underwent four-time gene duplication and one gene fusion event. Four-time gene duplication events of CA enzyme genes were indicated using the number in black circles. The gene fusion event was indicated using the number in the white circle. The colored blocks were used to represent the exons of CA enzyme genes. Same color indicates high sequence similarity. Exon segmentation of MI05G027520 gene was indicated using a lightning symbol. Bold characters represent putative C_4 genes.

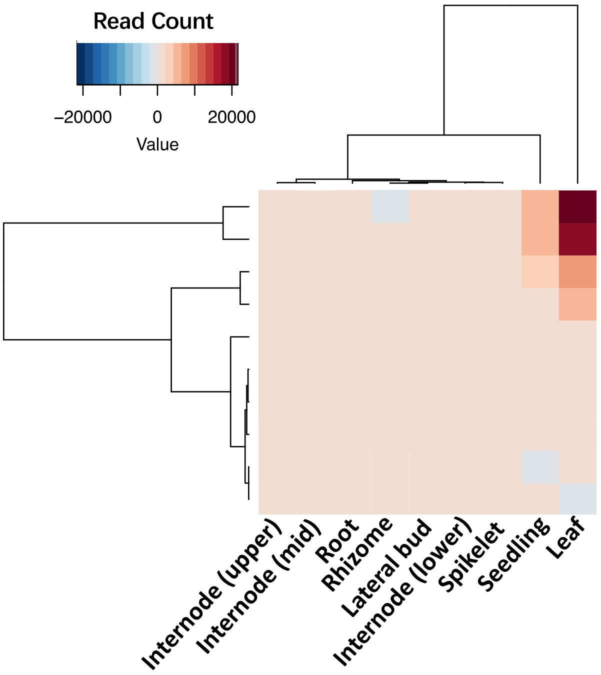

CA genes	GC	GC3s	ENC
**Ml06G028470**	**60.8%**	**85.0%**	**35.44**
**Ml05G027550**	**60.5%**	**84.6%**	**36.21**
**Ml05G027540**	**61.4%**	**85.1%**	**39.49**
**Ml06G028460**	**61.7%**	**86.2%**	**37.15**
Ml03G027080	56.2%	55.2%	61
Ml04G024880	50.2%	48.0%	59.96
Ml05G027530	63.7%	91.4%	32.39
Ml06G028450	63.7%	91.9%	30.91
Ml06G028440	52.5%	61.3%	56.91
Ml05G027520	51.7%	61.7%	53.41

Supplementary Fig. 40. Expression profile of *M. lutarioriparius* CA enzyme gene among different tissue samples and GC, GC3s and ENC of *M. lutarioriparius* CA enzyme genes. Different color gradients represent the library-normalized read count of each CA enzyme genes; GC3s: GC of silent 3rd codon position; ENC: The effective number of codons. Bold characters represent putative C_4 genes.

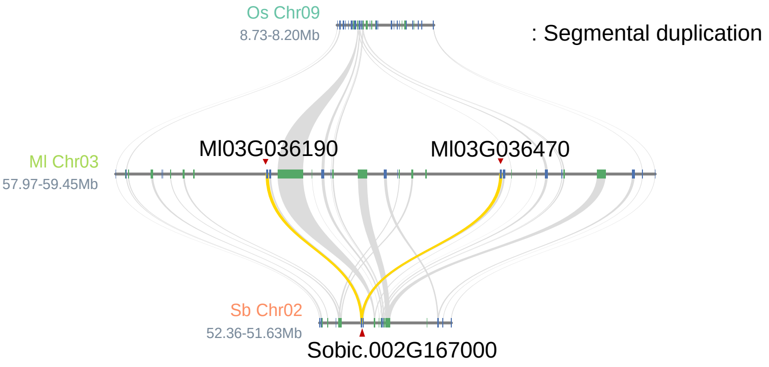

Supplementary Fig. 41. Gene synteny of *M. lutarioriparius*, sorghum and rice PEPC genes. Gene duplication of PEPC gene in *M. lutarioriparius* compared to sorghum.

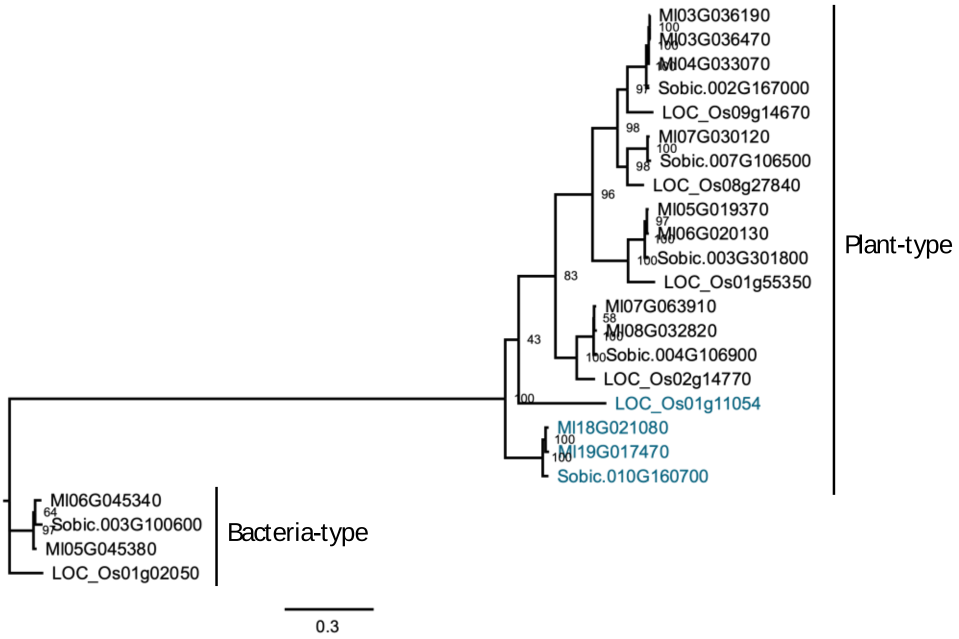

Supplementary Fig. 42. Maximum likelihood tree of *M. lutarioriparius*, sorghum and rice PEPC gene reconstructed using IQ-TREE software.

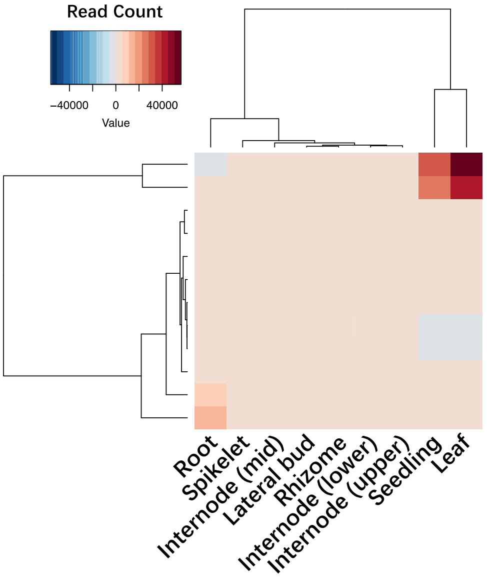

PEPC gene	GC	GC3s	ENC
**MI19G017470**	**61.2%**	**82.4%**	**40.84**
**MI18G021080**	**60.6%**	**81.3%**	**41.66**
MI05G019370	47.8%	45.4%	56.73
MI06G020130	47.7%	45.2%	56.43
MI04G033070	51.4%	54.8%	55.75
MI03G036470	51.2%	54.5%	55.72
MI07G030120	49.6%	49.1%	52.81
MI05G045380	55.5%	61.2%	55.99
MI06G045340	55.7%	62.8%	55.14
MI03G036190	51.4%	55.4%	55.5
MI08G032820	51.6%	52.9%	56.43
MI07G063910	51.8%	53.6%	56.39

Supplementary Fig. 43. The heatmap of transcriptome expression of *M. lutarioriparius* PEPC genes among nine transcriptome samples and GC, GC3s and ENC of *M. lutarioriparius* PEPC genes. Bold characters represent putative C_4 genes.

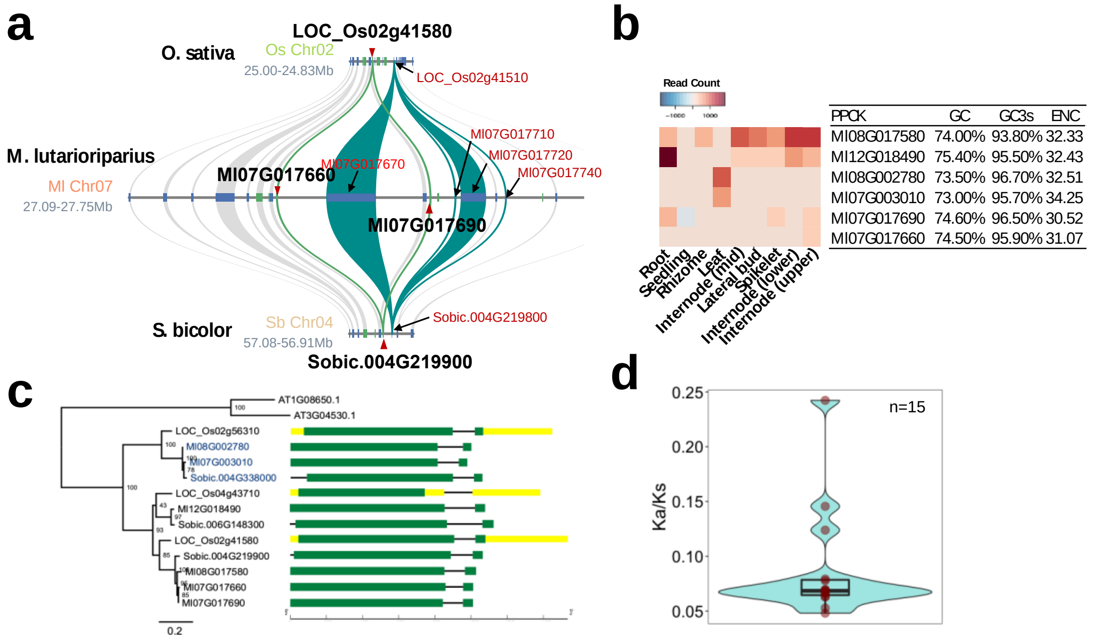

PPCK	GC	GC3s	ENC
Ml08G017580	74.00%	93.80%	32.33
Ml12G018490	75.40%	95.50%	32.43
Ml08G002780	73.50%	96.70%	32.51
Ml07G003010	73.00%	95.70%	34.25
Ml07G017690	74.60%	96.50%	30.52
Ml07G017660	74.50%	95.90%	31.07

Supplementary Fig. 44. PPCK genes of *M. lutarioriparius*. a Gene synteny of PPCK genes among *M. lutarioriparius*, sorghum and rice. Black gene symbols represent PPCK genes. **b** The expression heatmap of *M. lutarioriparius* PPCK genes among nine transcriptome samples, with information of GC, GC3s and ENC. **c** Molecular phylogenetic tree and gene structure diagram of PPCK genes of *M. lutarioriparius* and sorghum. Green rectangles and black lines represent exons and introns, respectively. **d** Boxplot of Ka/Ks of *M. lutarioriparius* PPCK genes. In boxplot, the center line indicates the median, the lower and upper hinges represent the first and third quartiles, the upper whisker extends to the largest value less than 1.5× the interquartile range (IQR), the lower whisker extends to the smallest value at most 1.5× the IQR, the red points represent the data points. The number of data points used for plotting is 15.

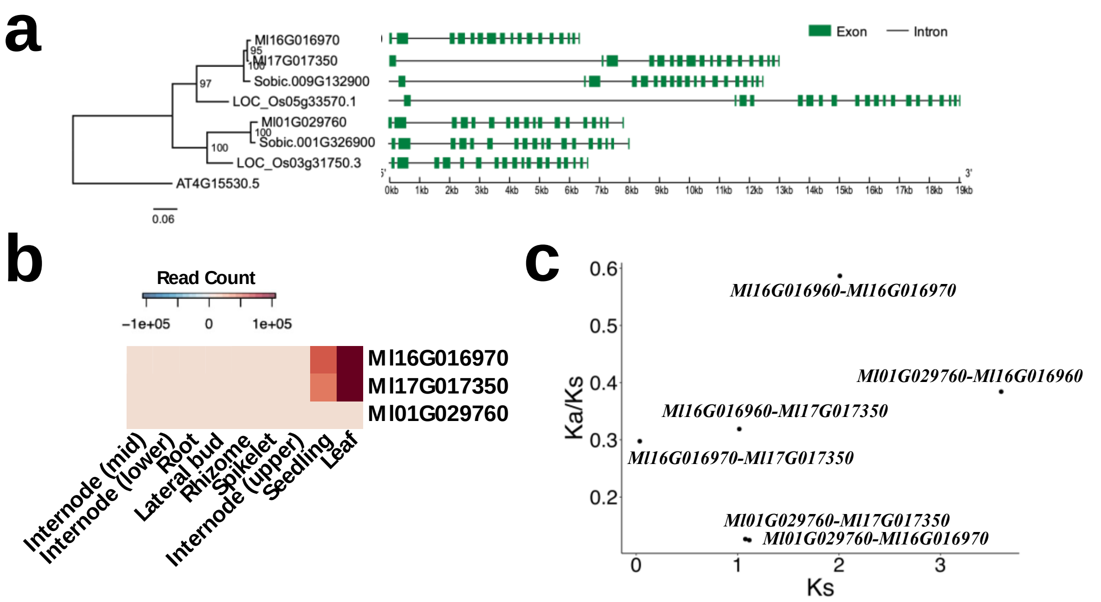

Supplementary Fig. 45. PPDK genes of *M. lutarioriparius*. a Molecular phylogenetic tree and gene structure diagram of PPDK genes of *M. lutarioriparius* and sorghum. Green

rectangles and black lines represent exons and introns, respectively. **b** The expression heatmap of *M. lutarioriparius* PPDK genes among nine transcriptome samples. **c** Distribution of *Ka/Ks* and *Ks* for *M. lutarioriparius* PPCK genes.

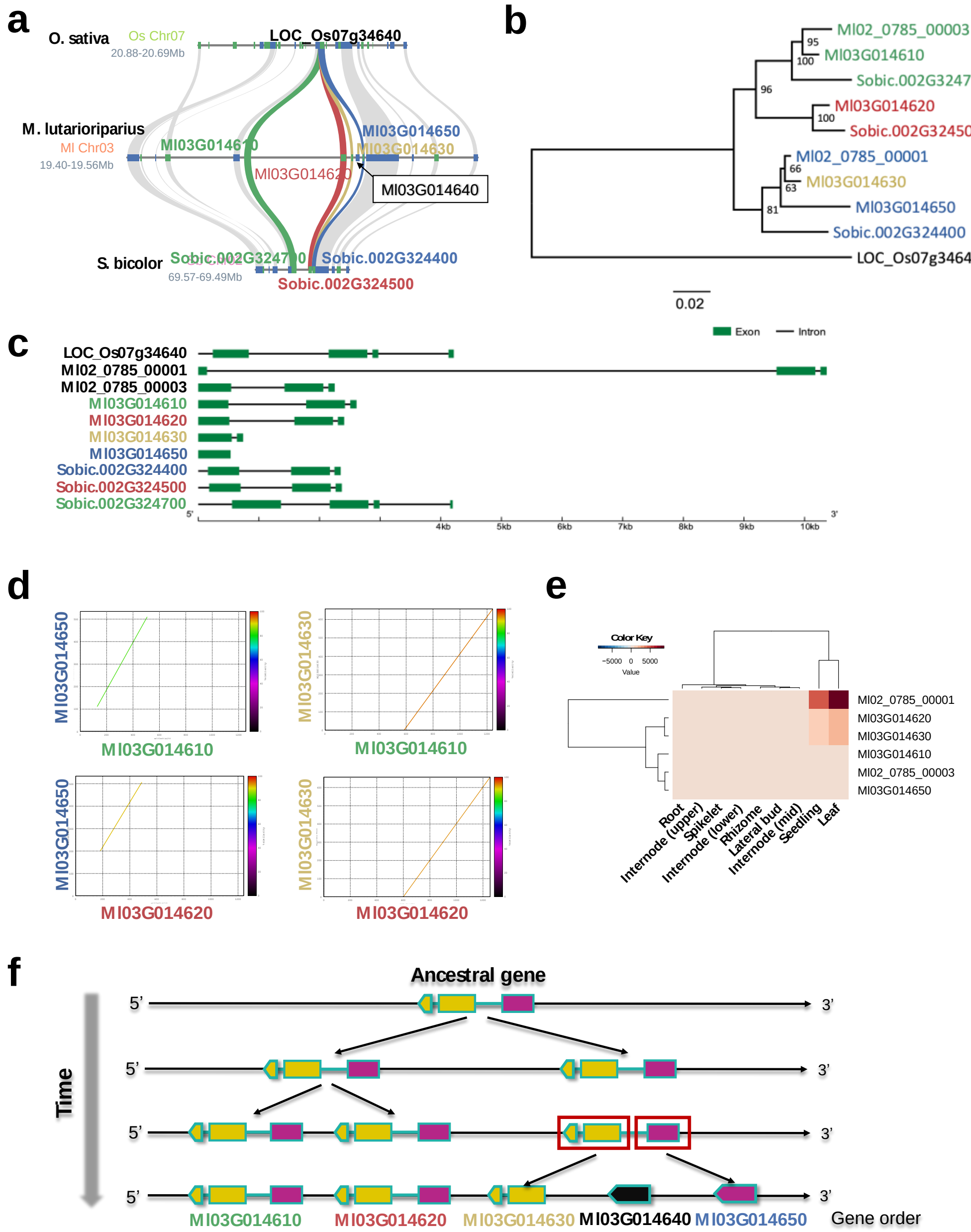

Supplementary Fig. 46. PPDK-RP genes of *M. lutarioriparius.* **a** Tandem duplications of *M. lutarioriparius* PPDK-RP genes. Ml03G014640 isn't a PPDK-RP gene. **b** Phylogenetic tree of *M. lutarioriparius* and sorghum PPDK-RP genes were reconstructed using Maximum likelihood (ML) approach. Numbers at nodes indicate the percentage bootstrap scores from 1,000 replicates. **c** Gene structure diagram of *M. lutarioriparius* and sorghum PPDK-RP genes. Green rectangles and black lines represent exons and introns, respectively. **d** Sequence comparisons of *M. lutarioriparius* PPDK-RP genes. **e** The expression heatmap of PPDK-RP genes among nine transcriptome samples. **f** Inferred duplication model of *M. lutarioriparius* PPDK-RP genes.

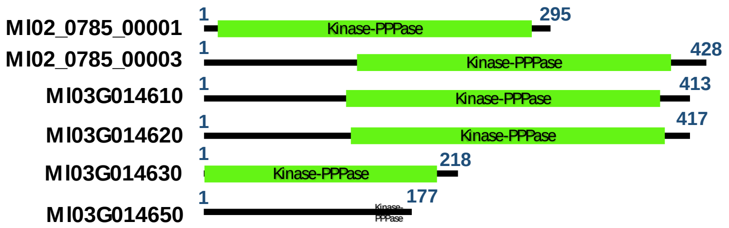

Supplementary Fig. 47. Functional domain annotation of *M. lutarioriparius* PPDK-RP genes

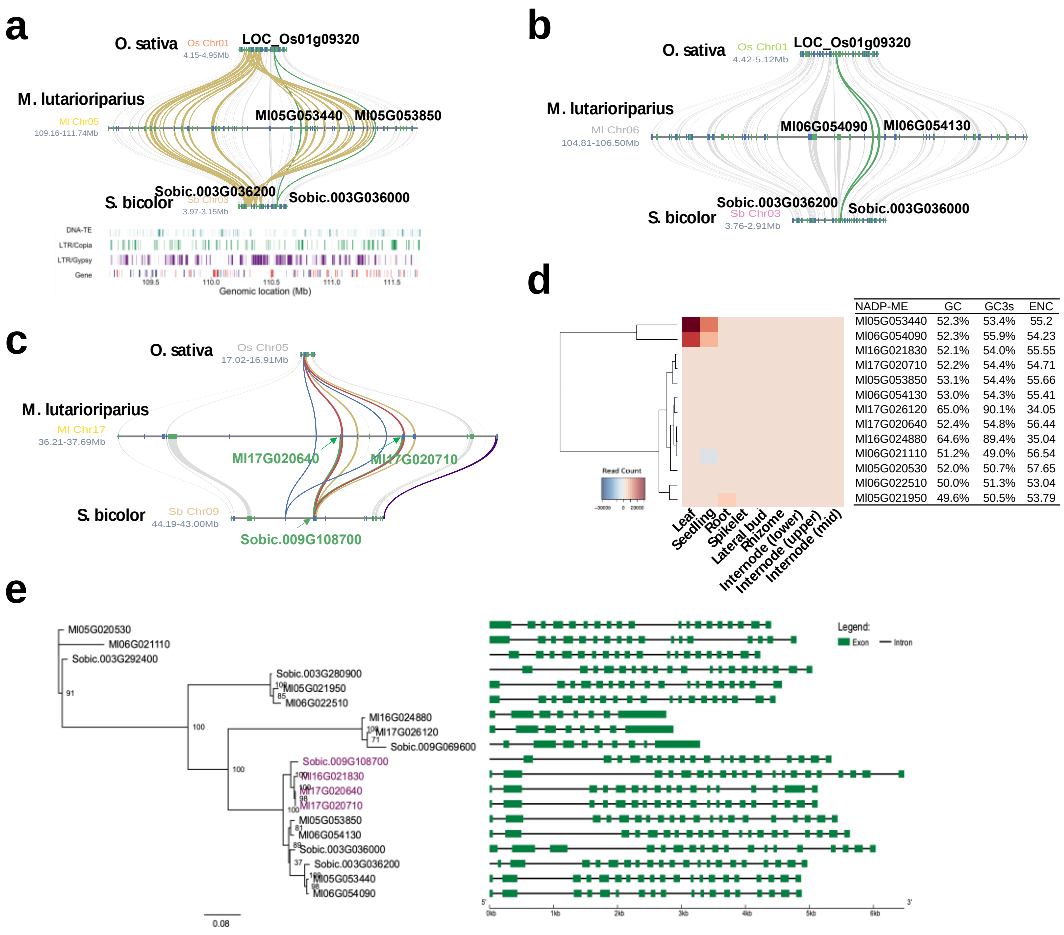

Supplementary Fig. 48. NADP-ME genes in *M. lutarioriparius*. a Tandem duplication of *M. lutarioriparius* NADP-ME genes Ml05G053440 and Ml05G053850. **b** Tandem duplication of *M. lutarioriparius* NADP-ME genes Ml06G054090 and Ml06G054130. **c** Segmental duplication of NADP-ME genes Ml17G020640 and Ml17G020710 only in *M. lutarioriparius*. **d** The expression heatmap of NADP-ME gene among 9 transcriptome samples, with the information of gene GC content, GC3s content and ENC. **e** Molecular phylogenetic tree and gene structure diagram of NADP-ME genes of *M. lutarioriparius* and sorghum.

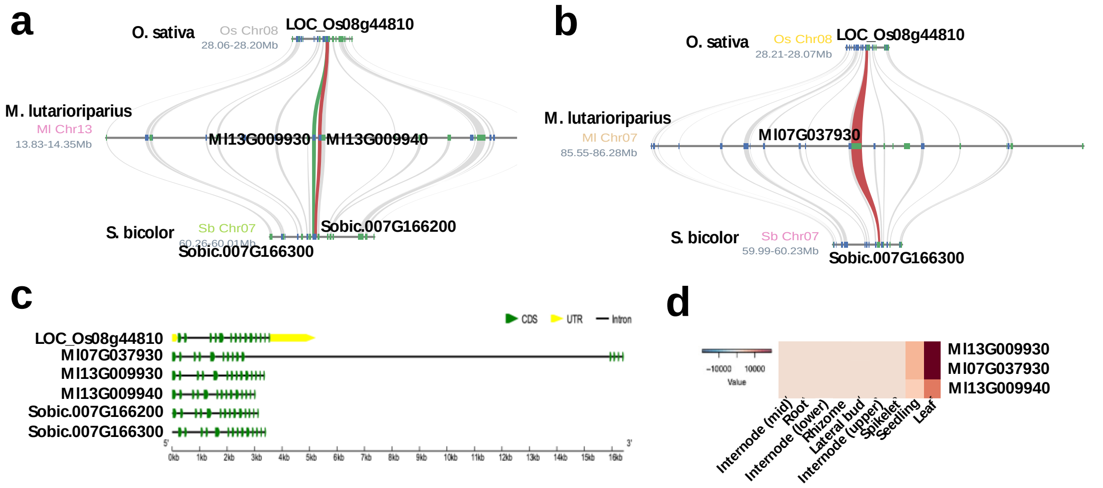

Supplementary Fig. 49. NADP-MDH genes in *M. lutarioriparius.* **a** Tandem duplication of *M. lutarioriparius* NADP-MDH genes Ml13G009930 and Ml13G009940. **b** Collinearity analysis of NADP-MDH gene Ml07G037930. **c** Gene structure diagram of *M. lutarioriparius*, rice and sorghum NADP-MDH genes. The green rectangles and black lines represent exons and introns, respectively. **d** Expression heatmap of NADP-MDH genes among nine transcriptome samples

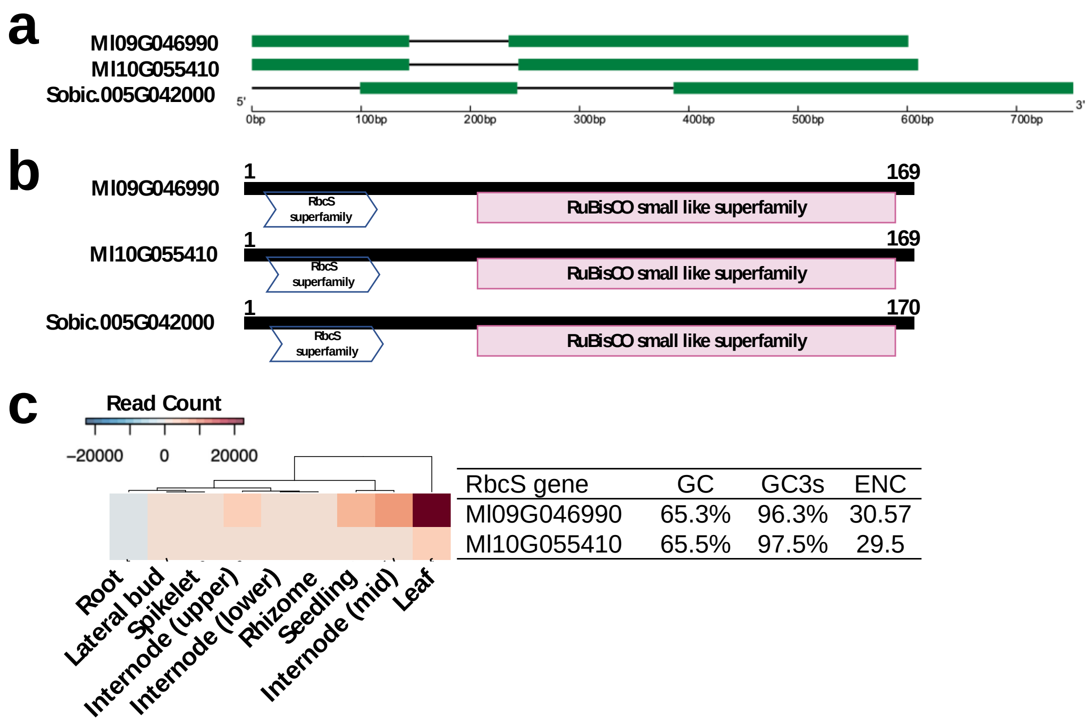

RbcS gene	GC	GC3s	ENC
Ml09G046990	65.3%	96.3%	30.57
Ml10G055410	65.5%	97.5%	29.5

Supplementary Fig. 50. RbcS genes in *M. lutarioriparius*. **a** Gene structure of *M. lutarioriparius* and sorghum RbcS genes. The green rectangle and black lines represent exons and introns respectively. **b** The functional domain annotation of *M. lutarioriparius* and sorghum RbcS genes. **c** Expression heatmap of *M. lutarioriparius* RbcS genes among nine transcriptome samples, with information of GC content, GC3s and ENC of *M. lutarioriparius* RbcS genes.

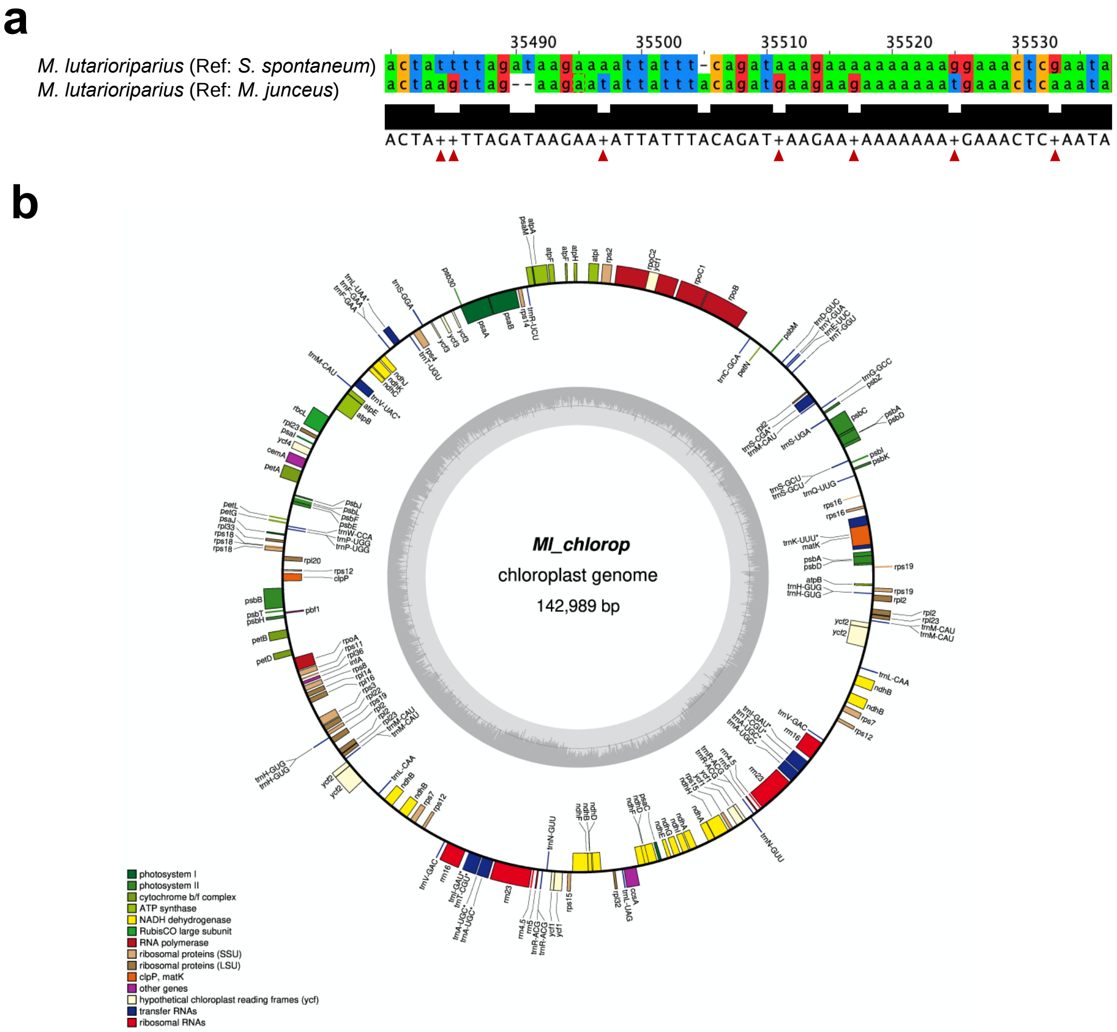

Supplementary Fig. 51. Visualization of annotation of *M. lutarioriparius* chloroplast genome assembled in this study. a Sequence comparison between two chloroplast genome assemblies of our sequencing accession. These two assemblies were reconstructed using the Illumina paired-end reads derived from whole genome sequencing through a baiting and iterative mapping approach, by which two references (*M. junceus* and *S. spontaeum*) were used. There are seven SNPs and one InDels between two *M. lutariroiparius* chloroplast genome assemblies. **b** The visualization of genome annotation of *M. lutarioriparius*.

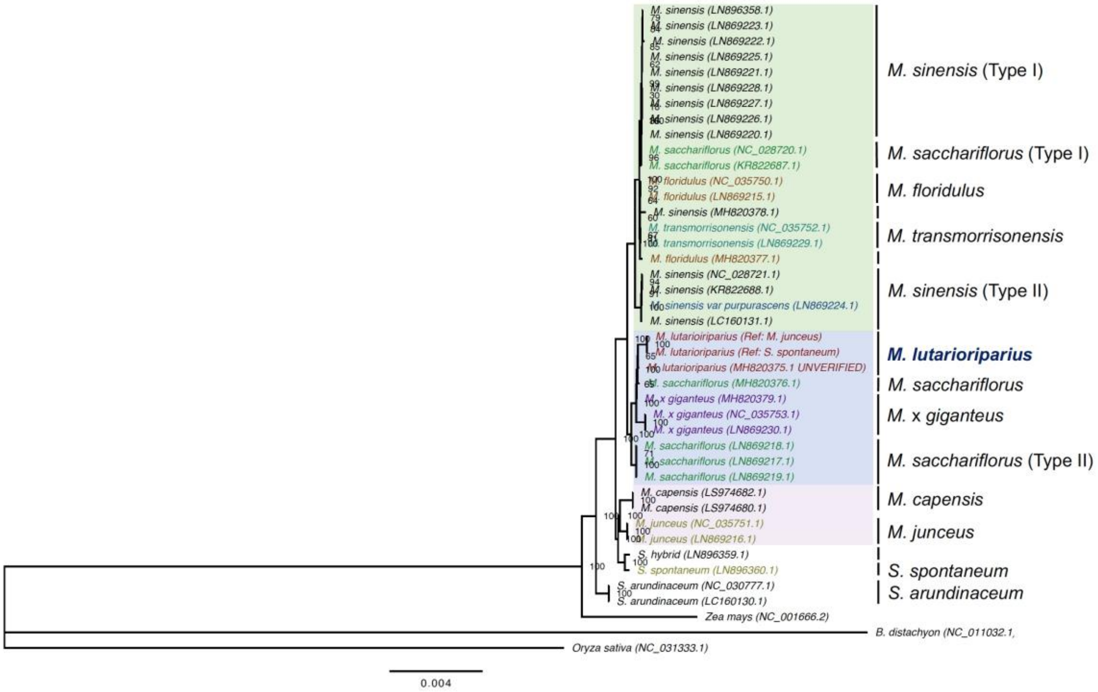

Supplementary Fig. 52. Phylogenetic tree of genus *Miscanthus* reconstructed using Maximum Likelihood (ML) approach based on whole chloroplast genome sequence. The chloroplast genome sequences were aligned using MAFFT[4] and the conserved sequence blocks were extracted using Gblock. The ML tree was reconstructed using IQ-TREE software with automatic best-fit model selection. The values of bootstrap support from 1,000 bootstrap replicates using IQ-TREE ultrafast bootstrap algorithm were indicated at nodes.

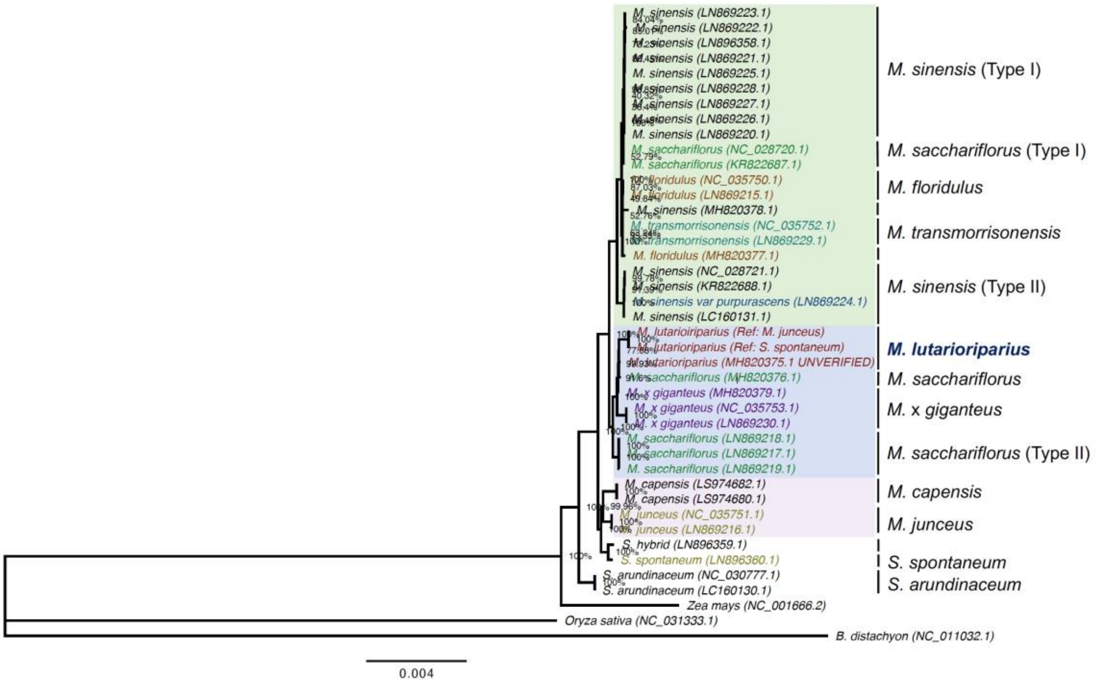

Supplementary Fig. 53. Phylogenetic tree of genus *Miscanthus* reconstructed using Neighbor-Joining (NJ) method based on whole chloroplast genome sequence. The chloroplast genome sequences were aligned using MAFFT[4] and the conserved sequence blocks were extracted using Gblock. The NJ tree was reconstructed using MEGA X[5] and 1,000 replicates bootstrap test were carried out. The values of bootstrap were indicated at nodes.

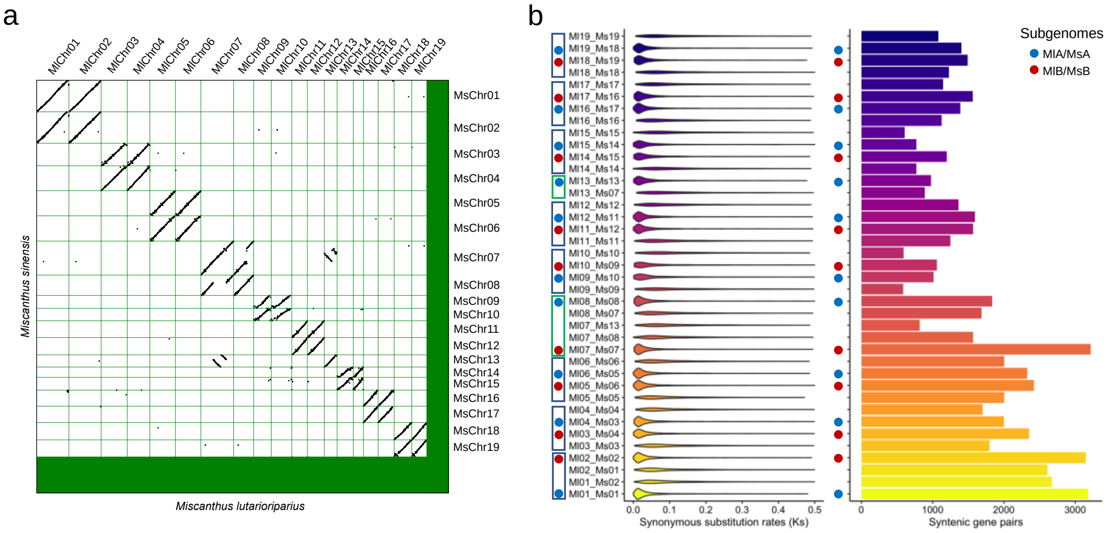

Supplementary Fig. 54. Gene synteny analysis for *Miscanthus lutarioriparius* and *Miscanthus sinensis*. a Gene synteny analysis for *Miscanthus lutarioriparius* and *Miscanthus sinensis*. **b** Violin plot of synonymous substitution rates of syntenic gene pairs of homoeologous chromosome pairs of *Miscanthus lutarioriparius* and *Miscanthus sinensis*. The right panel is the barplot of number of syntenic gene pairs of homologues chromosome pairs of *Miscanthus lutarioriparius* and *Miscanthus sinensis.*

Supplementary Table 1. Genome survey by k-mer method.

Item	Value
raw_peak	23
now_node	767,314,997
low_kmer	1,300,729,763
now_kmer	49,801,585,753
cvg	23
a[1/2]	0.0427106
a[1]	0.216474
b[1/2]	0.168431
b[1]	0.158596
Genome size (bp)	2,191,630,000
Repeat content	0.673
Heterozygosity	0.0013

Supplementary Table 2. Statistics of raw and clean Nanopore sequencing data.

Items	Raw data	Clean data
Number of sequences	13,992,488	10,037,103
Sum of bases	307,706,716,149	280,844,554,363
N50 (bp)	32,212	32,864
N90 (bp)	17,739	19,801
Mean sequence length (bp)	21,990	27,980
Maximum sequence length (bp)	250,629	250,629
Mean quality	6.81	7.96

Supplementary Table 3. The length distribution of clean Nanopore sequencing data.

Length (bp)	Reads number	Total Length (bp)	Percent (%)	Average Length (bp)
2,000~5,000	526,584	1,813,915,139	0.64	3,444.68
5,000~10,000	802,117	5,968,453,124	2.12	7,440.87
10,000~20,000	1,384,774	21,066,209,829	7.50	15,212.74
20,000~30,000	3,367,586	85,458,560,306	30.42	25,376.80
30,000~40,000	2,125,491	72,713,391,709	25.89	34,210.16
40,000~50,000	1,016,773	45,314,556,965	16.13	44,567.03
50,000~60,000	527,975	28,660,643,807	10.20	54,284.09
60,000~70,000	190,746	12,221,818,866	4.35	64,073.78
70,000~80,000	61,223	4,534,169,242	1.61	74,059.89
>=80,000	33,834	3,092,835,376	1.10	91,412.05

Supplementary Table 4. Statistics of Illumina DNA sequencing data.

Library	Insert size (bp)	Read length (bp)	Raw data (Gb)	Clean data (Gb)	Sequence depth (X)	GC%
ND_run448	~400	150	82.86	67.59	~38	46
N500_run445	~472	250	83.51	73.34	~41	44
N800_run445	~630	250	39.37	31.59	~18	44

Supplementary Table 5. Statistics of the number of coding genes and InterProScan annotation for 19 pseudochromosomes.

Pseudo-chromosome	Length (bp)	Number of coding gene	Number of genes annotated by InterProScan
Chr01	144,624,297	5,347	4,949
Chr02	141,797,626	5,352	4,966
Chr03	122,330,789	4,322	3,975
Chr04	108,741,605	3,766	3,529
Chr05	119,689,306	4,236	3,934
Chr06	113,455,120	4,226	3,917
Chr07	150,811,276	5,429	5,048
Chr08	90,590,548	3,328	3,130
Chr09	115,207,000	2,902	2,629
Chr10	130,888,329	3,439	3,129
Chr11	75,828,887	2,579	2,399
Chr12	83,158,305	2,801	2,598
Chr13	74,599,310	2,129	1,977
Chr14	94,845,455	2,692	2,426
Chr15	61,782,722	1,673	1,525
Chr16	79,875,055	2,446	2,298
Chr17	78,232,433	2,650	2,495
Chr18	88,986,391	2,933	2,650
Chr19	81,019,333	2,492	2,243
Total	1,956,463,787	64,742	59,817
Percentage	94.30%	94.75%	87.54%

Supplementary Table 6. Sequence similarity of syntenic chromosomes.

Chromosome pair	Number of base matches (bp)	Alignment block length (bp)	Sequence similarity (%)
Chr01/Chr02	45,885,018	137,212,078	33.44
Chr03/Chr04	34,247,603	103,561,892	33.07
Chr05/Chr06	36,840,587	110,083,315	33.47
Chr07/Chr08/Chr13	51,482,594	159,271,341	32.32
Chr09/Chr10	36,841,772	112,667,930	32.70
Chr11/Chr12	25,346,765	78,024,381	32.49
Chr14/Chr15	17,924,374	56,611,164	31.66
Chr16/Chr17	23,439,445	72,410,653	32.37
Chr18/Chr19	24,733,803	76,402,799	32.37

Supplementary Table 7. Comparison of Hi-C anchoring results generated by LACHSIS and 3d-dna pipeline.

Software	3d-dna pipeline					LACHESIS
Mode	Haploid		Diploid			
Mis-join correction	iterative = 0	iterative = 2	iterative = 0	iterative = 2	iterative = 7	
Number of scaffolds	163	3,996	163	3,996	5,743	919
Total length (bp)	2,310,174,655	2,311,039,655	1,903,956,058	1,923,638,092	1,929,321,746	2,074,797,027
Minimum sequence length (bp)	1,000	1,000	1,000	1,000	1,000	16,938
Maximum sequence length (bp)	188,012,908	169,516,646	147,938,815	144,831,376	136,530,390	150,811,276
Average sequence length (bp)	14,172,851	578,338	11,680,712	481,391	335,943	2,257,668
N50 length (bp)	102,464,518	110,158,741	83,487,897	91,965,924	86,557,408	113,455,120
N90 length (bp)	58,123,791	88,371,468	52,571,489	69,999,508	12,483,367	74,599,310
GC content (%)	45.44	45.43	45.43	45.40	45.38	45.46
NoN (bp)	2,308,788,550	2,308,959,719	1,903,384,871	1,922,291,545	1,927,392,424	2,074,589,547
Proportion of longest 19 scaffolds (%)	88.038	94.431	87.842	93.926	87.817	94.297
Complete BUSCOs (C)		1337 (97.2%)	1335 (97.1%)	1336 (97.2%)	1332 (96.9%)	1339 (97.4%)
Complete and single-copy BUSCOs (S)		462 (33.6%)	550 (40.0%)	500 (36.4%)	532 (38.7%)	480 (34.9%)
Complete and duplicated BUSCOs (D)		875 (63.6%)	785 (57.1%)	836 (60.8%)	800 (58.2%)	859 (62.5%)
Fragmented BUSCOs (F)		8 (0.6%)	9 (0.7%)	7 (0.5%)	8 (0.6%)	5 (0.4%)
Missing BUSCOs (M)		30 (2.2%)	31 (2.2%)	32 (2.3%)	35 (2.5%)	31 (2.2%)
LTR Assembly Index (LAI)		7.56		9.06	7.99	12.11
Illumina PE overall mapping rate (%)		99.94	99.77	99.76	99.74	99.82
Illumina PE properly paired rate (%)		97.43	95.51	95.52	95.50	96.28

Supplementary Table 8. Alignment summary of mRNA-Seq data.

Tissues	Total reads pairs	Overall alignment rate (%)	Read pairs aligned concordantly exactly 1 time	Read pairs aligned concordantly >1 time	Read pairs aligned concordantly 0 time
Rhizome	57,557,879	94.99	41,524,903 (72.14%)	11,434,473 (19.87%)	4,598,503 (7.99%)
Bud	46,439,831	94.83	35,468,029 (76.37%)	7,074,581 (15.23%)	3,897,221 (8.39%)
Internode (lower)	31,172,738	95.37	24,948,230 (80.03%)	4,114,803 (13.20%)	2,109,705 (6.77%)
Leaf	20,935,960	82.55	8,655,418 (41.34%)	6,266,168 (29.93%)	6,014,374 (28.73%)
Internode (mid)	31,490,989	95.35	25,280,984 (80.28%)	3,993,757 (12.68%)	2,216,248 (7.04%)
Root	29,081,420	93.01	21,844,282 (75.11%)	4,339,072 (14.92%)	2,898,066 (9.97%)
Seedling	14,920,573	89.78	6,288,476 (42.15%)	5,980,377 (40.08%)	2,651,720 (17.77%)
Spikelet	11,566,744	89.24	4,756,289 (41.12%)	5,169,620 (44.69%)	1,640,835 (14.19%)
Internode (upper)	31,285,582	95.25	24,913,488 (79.63%)	4,143,598 (13.24%)	2,228,496 (7.12%)

Supplementary Table 9. The average GC content among 8 species.

Species	Average GC content (%)	Genome size (Mb)
A. thaliana	36	119.67
B. distachyon	46.33	271.16
O. sativa	43.55	374.47
S. italica	45.60	405.74
Z. mays	46.20	2,135.08
S. bicolor	41.81	708.86
S. spontaneum	44.81	3,140.62
M. lutarioriparius	45.46	2,074.8

Supplementary Table 10. Chromosome position of potential centromeric regions.

Pseudo-chromosome	Start (bp)	End (bp)	Length (bp)
Chr01	74,439,407	76,426,273	1,986,866
Chr02	72,472,025	76,907,038	4,435,013
Chr03	68,117,061	69,229,757	1,112,696
Chr04	60,516,868	62,299,598	1,782,730
Chr05	65,512,690	66,263,644	750,954
Chr06	63,571,080	66,730,587	3,159,507
Chr07	60,512,231	61,250,233	738,002
Chr08	54,518,937	56,187,833	1,668,896
Chr09	51,573,634	59,132,349	7,558,715
Chr10	60,969,865	68,653,332	7,683,467
Chr11	54,544,432	55,417,084	872,652
Chr12	57,919,498	58,464,632	545,134
Chr13	39,266,917	40,054,122	787,205
Chr14	41,831,481	47,480,766	5,649,285
Chr15	27,631,199	35,271,239	7,640,040
Chr16	38,103,434	38,357,246	253,812
Chr17	44,674,089	45,750,082	1,075,993
Chr18	45,599,654	46,192,743	593,089
Chr19	38,912,793	39,019,645	106,852

Supplementary Table 11. Statistics of predicted gene models.

Item	Value
**Genes**	
Total length (bp)	270,509,919
Number of gene models	68,328
Mean gene length (bp)	3,959
Mean exon number per gene	4.77
Maximum exon number of genes	78
Number of single-exon gene	17,400
Number of genes annotated by InterProScan	63,076 (93.21%)
**CDSs**	
Total length (bp)	83,031,134
Number of CDS	68,328
Minimum CDS length (bp)	150
Maximum CDS length (bp)	17,073
Mean CDS length (bp)	1,215
Median CDS length (bp)	1,008
**Introns**	
Total length (bp)	187,547,029
Number of introns	257,898
Mean number per transcript	3.77
Mean intron length (bp)	727
Minimum intron length (bp)	21
Maximum intron length (bp)	199,504

Supplementary Table 12. BUSCO notation assessment of predicted genes.

Item	Value
Complete BUSCOs (C)	1,350 (98.2%)
Complete and single-copy BUSCOs (S)	375 (27.3%)
Complete and duplicated BUSCOs (D)	975 (70.9%)
Fragmented BUSCOs (F)	17 (1.2%)
Missing BUSCOs (M)	8 (0.6%)
Total BUSCO groups searched	1,375

Supplementary Table 13. Comparison of GC content of coding sequence among 8 species.

Species	CDS count	GC mean (%)	GC median (%)	GC3s mean (%)	GC3s median (%)
A. thaliana	27,416	44.45	44.20	40.53	39.80
B. distachyon	34,130	55.60	56.39	60.00	62.62
O. sativa	39,049	57.60	57.76	62.70	64.34
S. italica	34,584	56.50	57.43	62.60	65.01
Z. mays	39,498	54.80	56.31	60.00	63.18
S. bicolor	34,129	56.74	55.90	63.36	60.40
S. spontaneum	112,788	56.40	57.03	61.70	63.68
M. lutarioriparius	68,328	56.40	57.32	63.30	65.09

Supplementary Table 14. Statistics of repeat elements.

	Number of elements	Length occupied (bp)	Genome proportion (%)
LINEs	47,965	25,046,979	1.21
SINEs	20,278	3,383,723	0.16
LTR elements	634,807	970,568,697	46.78
DNA elements	495,461	200,045,511	9.64
Unclassified	396,649	136,871,521	6.6
Total interspersed repeats	1,595,160	1,335,916,431	64.39
Satellites	2,560	18,683,054	0.9

Supplementary Table 15. Statistics of MITEs.

MITEs	Value
Total length (bp)	4,836,422
Genome proportion	0.23%
Mean length	253.72
Number of MITE	19,062
Maximum length (bp)	800
Minimum length (bp)	60

Supplementary Table 16. Statistics of tandem repeats.

Item	Value
Total length (bp)	77,598,536
Genome proportion (%)	3.740
Average length (bp)	149.812
Count	517,973
Maximum length (bp)	131,208
Minimum length (bp)	25

Supplementary Table 17. Cellulose synthase encoding genes in *M. lutarioriparius.*

Gene ID	CDSL (bp)	Sb syntenic gene	CDSL (bp)	Os syntenic gene
Ml01G046000	3,252	Sobic.001G224300	3,177	LOC_Os10g32980
Ml01G069060	3,090	Sobic.001G045700	3,243	LOC_Os03g59340
Ml01G069240	3,294		3,243	LOC_Os03g59340
Ml01G072610	3,273	Sobic.001G021500	3,273	LOC_Os03g62090
Ml02_0088_00001	3,189	Sobic.009G063400	3,222	LOC_Os05g08370
Ml02_0380_00007	3,183			
Ml02G045300	3,246	Sobic.001G224300	3,177	LOC_Os10g32980
Ml02G069830	3,258	Sobic.001G045700	3,243	LOC_Os03g59340
Ml02G072870	3,273	Sobic.001G021500	3,273	LOC_Os03g62090
Ml03G030460	3,159	Sobic.002G205500	3,150	LOC_Os09g25490
Ml03G043090	3,216	Sobic.002G118700	3,366	LOC_Os07g24190
Ml03G045870	3,309	Sobic.002G094600	3,303	LOC_Os07g14850
Ml04G028220	3,156	Sobic.002G205500	3,150	LOC_Os09g25490
Ml04G039710	3,264	Sobic.002G118700	3,366	LOC_Os07g24190
Ml04G043020	3,294			
Ml04G043640	3,246	Sobic.002G075500	3,246	LOC_Os07g10770
Ml05G020050	2,949	Sobic.003G296400	2,943	LOC_Os01g54620
Ml05G051870	3,225			
Ml06G020630	2,946	Sobic.003G296400	2,943	LOC_Os01g54620
Ml06G052370	3,222			
Ml16G025580	2,808	Sobic.009G063400	3,222	LOC_Os05g08370
Ml17G026950	3,219	Sobic.009G063400	3,222	LOC_Os05g08370
Ml18G014840	5,616			
Ml18G015060	3,204			
Ml18G017550	2,592	Sobic.010G183700	2,649	LOC_Os06g39970
Ml19G012920	3,204			
Ml19G014510	2,433	Sobic.010G183700	2,649	LOC_Os06g39970

CDSL: coding sequence length. Sb: *Sorghum bicolor*. Os: *Oryza sativa*.

Supplementary Table 18. Comparative numbers of *CesA* and *Csl* gene families of *M. lutarioriparius*, maize, rice and *Arabidopsis.*

Gene	*M. lutarioriparius*	*O. sativa*	*Z. mays*	*A. thaliana*
CslA	18	9	10	9
CslB	0	0	0	6
CslC	14	6	8	5
CslD	16	5	5	5
CslF	27	9	7	0
CslH	5	3	0	0
CslG/E	10	3	3	4
CesA	27	11	20	9
Total	117	46	53	38

The data of gene count of sorghum, maize, rice and *Arabidopsis* was derived from cell wall genomics (https://cellwall.genomics.purdue.edu/families/index.html).

Supplementary Table 19. Comparative numbers of gene families involved in photosynthesis between *M. lutarioriparius* and sorghum.

Genes	Name	*M. lutarioriparius*	*S. bicolor*[6]
CA	Carbonic anhydrase	10 (4)	5 (2)
PEPC	phosphoenolpyruvate carboxylase	12 (2)	6 (1)
PPCK	Phosphoenolpyruvate carboxylase kinase	6 (2)	3 (1)
PPDK	Pyruvate phosphate dikinase	3 (2)	2 (1)
PPDK-RP	Pyruvate phosphate dikinase regulatory protein	6 (3)	3 (1)
NADP-ME	NADP-Malic enzyme	13 (2)	6 (1)
NADP-MDH	NADP-Malate dehydrogenase	3 (2)	2 (1)
RbcS	RbBPCase small-subunit	2 (1)	1 (1)

The number in the parentheses indicates the putative C_4 genes in that gene family.

Supplementary Table 20. CA enzyme genes information.

CA enzyme gene	GL (bp)	CDSL (bp)	Exon num.	Sb collinear gene	GL (bp)	CDSL (bp)	Os collinear gene
Ml03G027080	4,969	1,446	10	Sobic.002G230100	4,823	1,014	LOC_Os09g28910
Ml04G024880	3,071	702	7	Sobic.002G230100	4,823	1,014	LOC_Os09g28910
**Ml05G027550**	7,553	1,338	13	**Sobic.003G234200**	10,440	1,371	LOC_Os01g45274
**Ml06G028470**	6,672	1,338	13	**Sobic.003G234200**	10,440	1,371	LOC_Os01g45274
**Ml05G027540**	4,859	609	7	**Sobic.003G234400**	4,749	615	LOC_Os01g45274
**Ml06G028460**	4,459	702	7	**Sobic.003G234400**	4,749	615	LOC_Os01g45274
Ml05G027530	2,207	609	6	Sobic.003G234500	2,986	609	LOC_Os01g45274
Ml06G028450	2,259	609	6	Sobic.003G234500	2,986	609	LOC_Os01g45274
Ml05G027520	3,252	747	8	Sobic.003G234600	4,750	771	LOC_Os01g45274
Ml06G028440	3,719	753	7	Sobic.003G234600	4,750	771	LOC_Os01g45274

CDSL: coding sequence length. GL: gene length. Sb: *Sorghum bicolor*. Os: *Oryza sativa*. Bold characters represent putative C_4 genes.

Supplementary Table 21. Information of PEPC enzyme gene in *M. lutarioriparius*.

PEPC gene	GL (bp)	CDSL (bp)	Exon num.	Sb collinear gene	GL (bp)	CDSL (bp)	Os collinear gene
Ml03G036190	5,069	2,736	11	Sobic.002G167000	5,632	2,904	
Ml03G036470	5,072	2,889	11	Sobic.002G167000	5,632	2,904	
Ml04G033070	5,063	2,904	10	Sobic.002G167000	5,632	2,904	LOC_Os09g14670
Ml05G045380	8,760	2,946	18	Sobic.003G100600	8,881	3,117	LOC_Os01g02050
Ml06G045340	7,433	2,817	18	Sobic.003G100600	8,881	3,117	LOC_Os01g02050
Ml05G019370	11,863	2,901	10	Sobic.003G301800	7,610	2,901	LOC_Os01g55350
Ml06G020130	6,194	2,901	9	Sobic.003G301800	7,610	2,901	LOC_Os01g55350
Ml07G063910	6,706	2,883	10	Sobic.004G106900	6,977	2,883	LOC_Os02g14770
Ml08G032820	6,719	2,883	10	Sobic.004G106900	6,977	2,883	LOC_Os02g14770
Ml07G030120	4,927	2,874	10	Sobic.007G106500	5,616	2,895	LOC_Os08g27840
**Ml18G021080**	7,426	2,889	10	**Sobic.010G160700**	6,647	3,087	LOC_Os01g11054
**Ml19G017470**	7,673	2,886	10	**Sobic.010G160700**	6,647	3,087	LOC_Os01g11054

CDSL: coding sequence length. GL: gene length. Sb: *Sorghum bicolor*. Os: *Oryza sativa*. Bold characters represent putative C_4 genes.

Supplementary Table 22. Information of *M. lutarioriparius* PPCK genes.

PPCK	GL (bp)	CDSL (bp)	Exon num.	Sb collinear gene	GL (bp)	CDSL (bp)	Os collinear gene
**Ml07G003010**	978	861	2	**Sobic.004G338000**	1,749	855	LOC_Os02g56310
Ml07G017660	1,012	915	2	Sobic.004G219900	1,612	924	LOC_Os02g41580
Ml07G017690	1,011	900	2	Sobic.004G219900	1,612	924	LOC_Os02g41580
Ml08G017580	1,028	915	2	Sobic.004G219900	1,612	924	LOC_Os02g41580
**Ml08G002780**	1,001	861	2	**Sobic.004G338000**	1,749	855	LOC_Os02g56310
Ml12G018490	1,080	912	2	Sobic.006G148300	1,997	900	LOC_Os04g43710

CDSL: coding sequence length. GL: gene length. Sb: *Sorghum bicolor*. Os: *Oryza sativa*. Bold characters represent putative C4 genes.

Supplementary Table 23. Information of *M. lutarioriparius* PPDK genes.

PPDK	GL (bp)	CDSL (bp)	Exon num.	Sb collinear gene	GL (bp)	CDSL (bp)	Os colinear gene
Ml01G029760	7,855	2,703	18	Sobic.001G326900	8,494	2,730	LOC_Os03g31750
**Ml16G016970**	6,354	2,649	17	**Sobic.009G132900**	12,748	2,847	LOC_Os05g33570
**Ml17G017350**	13,017	2,844	18	**Sobic.009G132900**	12,748	2,847	LOC_Os05g33570

CDSL: coding sequence length. GL: gene length. Sb: *Sorghum bicolor*. Os: *Oryza sativa*. Bold characters represent putative C_4 genes.

Supplementary Table 24. Information of *M. lutarioriparius* PPDK-RP genes.

PPDK-RP	GL (bp)	CDSL (bp)	Exon num.	Sorghum ortholog	GL (bp)	CDSL (bp)	Rice ortholog
**Ml02_0785_00001**	10,365	888	3	**Sobic.002G324400**	2,507	1,290	LOC_Os07g34640
Ml02_0785_00003	2,251	1,287	3	Sobic.002G324700	4,662	1,587	LOC_Os07g34640
Ml03G014610	2,611	1,242	3	Sobic.002G324700	4,662	1,587	LOC_Os07g34640
Ml03G014620	2,407	1,254	3	Sobic.002G324500	3,072	1,260	LOC_Os07g34640
**Ml03G014630**	740	657	2	**Sobic.002G324400**	2,507	1,290	LOC_Os07g34640
**Ml03G014650**	533	534	1	**Sobic.002G324400**	2,507	1,290	LOC_Os07g34640

CDSL: coding sequence length. GL: gene length. Bold characters represent putative C_4 genes.

Supplementary Table 25. The information of *M. lutarioriparius* NADP-ME genes.

NADP-ME	GL (bp)	CDSL (bp)	Exon number	Sorghum collinear gene	GL (bp)	CDSL (bp)
Ml05G053850	8,365	1,950	20	Sobic.003G036000	6,107	2,124
Ml06G054130	15,675	1,929	20	Sobic.003G036000	6,107	2,124
**Ml05G053440**	6,412	1,908	20	**Sobic.003G036200**	5,447	1,911
**Ml06G054090**	13,947	1,686	18	**Sobic.003G036200**	5,447	1,911
Ml05G021950	2,541	1,698	18	Sobic.003G280900	5,691	1,782
Ml06G022510	121,22	1,782	19	Sobic.003G280900	5,691	1,782
Ml05G020530	4,401	1,962	19	Sobic.003G292400	4,527	1,782
Ml06G021110	10,361	1,719	17	Sobic.003G292400	4,527	1,782
Ml16G024880	21,405	1,713	8	Sobic.009G069600	3,624	1,713
Ml17G026120	28,537	1,713	8	Sobic.009G069600	3,624	1,713
Ml16G021830	19,406	1,953	20	Sobic.009G108700	5,651	1,959
Ml17G020640	24,680	1,857	17	Sobic.009G108700	5,651	1,959
Ml17G020710	26,581	1,911	20	Sobic.009G108700	5,651	1,959

CDSL: coding sequence length. GL: gene length. Bold characters represent putative C_4 genes.

Supplementary Table 26. The information of *M. lutarioriparius* NADP-MDH genes.

NADP-MDH	CDSL (bp)	Exon num.	GL (bp)	Sb collinear gene	GL (bp)	CDSL (bp)	Os collinear gene
Ml13G009940	1,314	14	3,041	Sobic.007G166200	3,354	1,308	LOC_Os08g44810
**Ml07G037930**	1,302	14	16,434	**Sobic.007G166300**	3,816	1,290	LOC_Os08g44810
**Ml13G009930**	1,302	14	3,378	**Sobic.007G166300**	3,816	1,290	LOC_Os08g44810

CDSL: coding sequence length. GL: gene length. Sb: *Sorghum bicolor.* Os: *Oryza sativa*. Bold characters represent putative C_4 genes.

Supplementary Table 27. The quality statistic of Hi-C data.

Item	Value	Percent
Total read pairs processed	1,159,214,515	100.00%
Unmapped read pairs	24,633,679	2.13%
Low quality read pairs	0	0.00%
Unique paired alignments	50,949,808	43.95%
Multiple paired alignments	49,628,487	42.81%
Pairs with singleton	128,797,873	11.11%
Low quality singleton	0	0.00%
Unique singleton alignments	0	0.00%
Multiple singleton alignments	0	0.00%
Reported pairs	509,498,087	43.95%

Supplementary Table 28. Statistics of valid interaction read pairs in Hi-C data.

Item	Value	Percent
Valid interaction pairs	371,731,170	32.07%
Valid interaction rmdup	282,204,751	24.34%
Trans interaction	187,647,612	16.19%
Cis interaction	94,557,139	8.16%
Cis short-range interaction	37,614,554	3.24%
Cis long-range interaction	56,942,585	4.91%

Supplementary Note 1. Evolution of C4 photosynthesis genes in *M. lutarioriparius*.

Carbonic anhydrase genes

Ten Carbonic anhydrase (CA) genes were identified in *M. lutarioriparius* genome (**Supplementary Fig. 34, 35** and **Supplementary Table 20**), among which, eight CA genes are located in two tandem duplication blocks (**Supplementary Fig. 34a, b)**. Based on sequence similarity and gene structure, Ml05G027550/Ml06G028470 was inferred to be derived from the fusion of two neighboring CA genes, which probably occurred before the recent whole genome duplication event (WGD) (**Supplementary Fig. 34c and 36**). To rule out the possibility that two neighboring CA genes were incorrectly annotated as one fused CA gene by gene prediction, transcriptome data of leaf sample was used to verify the gene fusion event. Transcriptome reads from leaf samples support the gene fusion event happened in CA gene family of *M. lutarioriparius* (**Supplementary Fig. 37**). Ml04G024880 seem to be truncated compared with the homolog Ml03G027080 (**Supplementary Fig. 35c, d)**. Tandem duplication plays a critical role in the evolution of CA enzyme genes in *M. lutarioriparius*. Based on the phylogeny and genomic information (**Supplementary Fig. 34 and 38**), the CA enzyme genes in *M. lutarioriparius* were inferred to undergo four-time single gene duplication events, one-time gene fusion event and the recent WGD, which made 10 copies (**Supplementary Fig. 39**). An exon segmentation of Ml05G027520 was probably occurred (**Supplementary Fig. 34c**). Transcriptome analysis showed that the four CA enzyme genes colinear with the sorghum C_4 CA genes had specific high expression in leaves, supporting these four gene probably be C_4 CA enzyme genes in *M. lutarioriparius* (**Supplementary Fig. 40**). Codon usages bias analysis showed that the four possible C_4 CA genes had high GC3s (84.6% ~ 86.2%) and low Nc (Effective number of codons), similar to that of sorghum C_4 CA genes (82.6% and 86.4%) (**Supplementary Fig. 40**).

Phosphoenolpyruvate carboxylase genes

Twelve phosphoenolpyruvate carboxylase (PEPC) genes were identified in *M. lutarioriparius*, which is more than that of sorghum (6) (**Supplementary Table 21**). Besides the gene family expansion resulting from the recent WGD, segmental duplication event occurred after the WGD further expanded the PEPC gene family in *M. lutarioriparius* (**Supplementary Fig. 41**). Phylogenetic analysis suggested all *M. lutarioriparius* PEPC genes could be divided into six distinct groups, which is in consistent with previous study[7]. In *M. lutarioriparius*, two PEPC genes were predicted to encode bacteria-type enzymes and ten to encode plant-type enzymes (**Supplementary Fig. 42**). Transcriptome data showed that the two bacteria-type PEPC genes has no expression in the leaf and very low

expression in other samples. While Ml18G021080 and Ml19G017470 have specific high expression in the leaf and seedling (**Supplementary Fig. 43**). In additional, Ml18G021080 and Ml19G017470 both are collinear with sorghum C_4 PEPC gene Sobic.010G160700. Condon usage analysis revealed that Ml18G021080 and Ml19G017470 has relatively higher GC3s (81.3% and 82.4%) among all PEPC genes, which is similar to that of sorghum C_4 PEPC. Combined with the above information, we inferred that Ml18G021080 and Ml19G017470 were the functional C_4 PEPC genes in *M. lutarioriparius*.

Phosphoenolpyruvate carboxylase kinase

Phosphoenolpyruvate carboxylase kinase (PPCK) is responsible for the phosphorylation of leaf PEPC during C_4 photosynthesis[8]. There were six PPCK genes identified in *M. lutarioriparius* (**Supplementary Table 22**). One tandem duplication of PPCK genes further expanded the PPCK gene family in *M. lutarioriparius* (**Supplementary Fig. 44a**). PPCK genes of *M. lutarioriparius*, rice and sorghum all have high GC3s (>91%) (**Supplementary Fig. 44b**). Transcriptome analysis suggested the putative C_4 PPCK genes Ml07G003010 and Ml08G002780 had specific high expression in leaf compared with other samples (**Supplementary Fig. 44b**). Phylogenetic analysis indicated the differentiation of C_4 and non-C_4 isoform PPCK probably occurred before species divergence among grass (**Supplementary Fig. 44c**). PPCK gene family probably suffer the purify selection due to the important function in C_4 photosynthesis (**Supplementary Fig. 44d**).

Pyruvate phosphate dikinase genes

Three genes coding pyruvate phosphate dikinase (PPDK) were identified in *M. lutarioriparius* genome (**Supplementary Table 23**). Ml16G016970 and Ml17G017350 both are collinear with the sorghum C_4 PPDK gene Sobic.009G132900[9]. And phylogenetic analysis revealed that putative C_4 isoform PPDK genes of *M. lutarioriparius* and sorghum were grouped in the same cluster (**Supplementary Fig. 45a**). Two C_4 isoform *M. lutarioriparius* PPDK genes both showed specific high expression in leaf and seedling compared to other samples (**Supplementary Fig. 45b**).

Pyruvate phosphate dikinase regulatory protein genes

Totally six pyruvate phosphate dikinase regulatory protein (PPDK-RP) genes were identified in *M. lutarioriparius*, among which, four are tandem duplicates (**Supplementary Table 24**). Based on the collinearity with sorghum C_4 PPDK-RP gene, three putative C_4 PPDK-RP genes of *M. lutarioriparius* were characterized. Transcriptome analysis revealed these three putative C_4 PPDK-RP genes highly expressed in leaf sample compared to other samples (**Supplementary Fig. 46e**). Based on the collinearity relationship of *M. lutarioriparius,* sorghum and rice, the tandem duplications of PPDK-RP genes were

inferred to be occurred before the species divergence between *M. lutarioriparius* and sorghum (**Supplementary Fig. 46a**). Combing phylogenetic analysis, gene structure and sequence similarity (**Supplementary Fig. 46a, b, c, d**), PPDK-RP genes in *M. lutarioriparius* were inferred to undergo two-time gene duplications and one-time gene fission event (**Supplementary Fig. 46f**). Function domain annotation indicated that Ml03G014630 and Ml03G014650 both had partial kinase-PPPase domain (**Supplementary Fig. 47**).

NADP-malic enzyme genes

We identified 13 NADP-malic enzyme (NADP-ME) genes in *M. lutarioriparius* (**Supplementary Table 25** and **Supplementary Fig. 48e**). The duplications of *M. lutarioriparius* NADP-ME occurred before and after it split with sorghum, leading to the expansion of size of NADP-ME gene family in *M. lutarioriparius* (**Supplementary Fig. 48a, b and c**). Transcriptome data showed that the putative C_4 NADP-ME genes in *M. lutarioriparius* had specific high expression in leaf compared to other samples (**Supplementary Fig. 48d**).

NADP-malate dehydrogenase genes

There exist two NADP-malate dehydrogenase (NADP-MDH) genes in sorghum genome, one is C_4 gene, the other is non-C_4 isoform gene. A total of three NADP-MDH genes of *M. lutarioriparius* were identified using homolog search (**Supplementary Table 26**). Based on the collinearity analysis, it was speculated that the tandem duplication of NADP-MDH genes of sorghum might occur after the species differentiation of sorghum and rice, but before the species differentiation of *M. lutarioriparius* and sorghum. Therefore, the tandem duplication of NADP-MDH gene was also observed in *M. lutarioriparius* genome. One copy of *M. lutarioriparius* NADP-MDH gene probably be lost after the recent WGD (**Supplementary Fig. 49a, b**). The results of subcellular localization prediction showed that the productions of three NADP-MDH genes were located in chloroplast. Transcriptome data analysis revealed that these three genes were all highly expressed in leaf tissues (**Supplementary Fig. 49d**). Ml07G037930 and Ml13G009940, which were collinear with the sorghum C_4 NADP-MDH gene, had higher expression levels than Ml13G009940, supporting Ml07G037930 and Ml13G009930 as potential C_4 NADP-MDH genes of *M. lutarioriparius* (**Supplementary Fig. 49d**). The analysis of codon usage revealed that similar GC3s (46.3%~49.8%) among *M. lutarioriparius* NADP-MDH genes and homologous genes of sorghum and rice.

RbBPCase small-subunit genes

The Localization of Rubisco in C_4 plant vascular sheath depends on the RbBPCase small-

subunit (RbcS) genes. Homologous genes were retrieved from the genome of *M. lutariroriparius* by using the known sorghum RbcS gene Sobic.005G042000, and two *M. lutariroriparius* RbcS genes were identified (**Supplementary Fig. 50a, b**). Codon analysis showed high GC3s of two RbcS genes (96.3% and 97.5%), similar to sorghum homologous gene Sobic.005G042000 (97.5%) (**Supplementary Fig. 50c**). Transcriptome data analysis revealed that both genes were expressed in leaves, but the expression level of Ml09G046990 gene in leaves was much higher than that of Ml10G055410, indicating that Ml09G046990 probably the gene mainly performing photosynthesis-related functions (**Supplementary Fig. 50c**).

Supplementary references

1. Wang, Y. *et al*. MCScanX: a toolkit for detection and evolutionary analysis of gene synteny and collinearity. *Nucleic Acids Res.* **40**, e49-e49 (2012).
2. Benjamini, Y. & Hochberg, Y. Controlling the false discovery rate: a practical and powerful approach to multiple testing. *J. R. Stat. Soc. Ser.* B **57**, 289-300 (1995).
3. Pinard, D. *et al*. Comparative analysis of plant carbohydrate active enzymes and their role in xylogenesis. *BMC Genomics* **16**, 402 (2015).
4. Katoh, K. & Standley, D. M. MAFFT multiple sequence alignment software version 7: Improvements in performance and usability. *Mol. Biol. Evol.* **30**, 772-780 (2013).
5. Kumar, S., Stecher, G., Li, M., Knyaz, C. & Tamura, K. MEGA X: molecular evolutionary genetics analysis across computing platforms. *Mol. Biol. Evol.* **35**, 1547-1549 (2018).
6. Wang, X. *et al.* Comparative genomic analysis of C4 photosynthetic pathway evolution in grasses. *Genome Biol.* **10**, R68 (2009).
7. Sánchez, R. & Cejudo, F. J. Identification and expression analysis of a gene encoding a bacterial-type phosphoenolpyruvate carboxylase from *Arabidopsis* and Rice. *Plant Physiol.* **132**, 949-957 (2003).
8. Aldous, S. H. *et al.* Evolution of the phosphoenolpyruvate carboxylase protein kinase family in C3 and C4 *Flaveria* spp. *Plant Physiol.* **165**, 1076-1091 (2014).
9. Paterson, A. H. *et al.* The *sorghum bicolor* genome and the diversification of grasses. *Nature* **457**, 551-556 (2009).
