# Supplementary material for: Chromosome-scale assembly and analysis of biomass crop Miscanthus lutarioriparius genome
Source: Nat Commun. 2021 Apr 28;12:2458. doi: 10.1038/s41467-021-22738-4 (PMC8080599; doi:10.1038/s41467-021-22738-4)
Supplement: Supplementary file 7 — Description of Additional Supplementary Files [file 41467_2021_22738_MOESM7_ESM.pdf]

Description of additional supplementary files

Title: Supplementary Data 1

Description: Statistic of NBSencoding genes.

Title: Supplementary Data 2

Description: Cellulose synthase-like encoding genes of *M. lutarioriparius*.

Title: Supplementary Data 3

Description: Statistics of lignin biosynthesis enzyme encoding genes.

Title: Supplementary Data 4

Description: Information of chloroplast genomes.
